# Loss of Endothelial Endoglin Promotes High-Output Heart Failure Through Peripheral Arteriovenous Shunting Driven by VEGF Signaling

**DOI:** 10.1161/CIRCRESAHA.119.315974

**Published:** 2019-12-06

**Authors:** Simon Tual-Chalot, Maria Garcia-Collado, Rachael E. Redgrave, Esha Singh, Benjamin Davison, Catherine Park, Hua Lin, Saimir Luli, Yi Jin, Yixin Wang, Allan Lawrie, Lars Jakobsson, Helen M. Arthur

**Affiliations:** 1 From the Biosciences Institute (S.T.-C., R.E.R., E.S., B.D., C.P., H.L., H.M.A.), Faculty of Medical Sciences, Newcastle University, United Kingdom; 2 Preclinical In Vivo Imaging Facility (S.L.), Faculty of Medical Sciences, Newcastle University, United Kingdom; 3 Karolinska Institutet, Solna, Sweden (M.G.-C., Y.J., Y.W., L.J.); 4 Department of Infection, Immunity and Cardiovascular Disease, University of Sheffield, United Kingdom (A.L.).

**Keywords:** arteriovenous malformations, endoglin, endothelial cells, hypoxia, vascular diseases

## Abstract

**Rationale:**

ENG (endoglin) is a coreceptor for BMP (bone morphogenetic protein) 9/10 and is strongly expressed in endothelial cells. Mutations in *ENG* lead to the inherited vascular disorder hereditary hemorrhagic telangiectasia characterized by local telangiectases and larger arteriovenous malformations (AVMs); but how ENG functions to regulate the adult vasculature is not understood.

**Objective:**

The goal of the work was to determine how ENG maintains vessel caliber in adult life to prevent AVM formation and thereby protect heart function.

**Methods and Results:**

Genetic depletion of endothelial *Eng* in adult mice led to a significant reduction in mean aortic blood pressure. There was no evidence of hemorrhage, anemia, or AVMs in major organs to explain the reduced aortic pressure. However, large AVMs developed in the peripheral vasculature intimately associated with the pelvic cartilaginous symphysis—a noncapsulated cartilage with a naturally high endogenous expression of VEGF (vascular endothelial growth factor). The increased blood flow through these peripheral AVMs explained the drop in aortic blood pressure and led to increased cardiac preload, and high stroke volumes, ultimately resulting in high-output heart failure. Development of pelvic AVMs in this region of high VEGF expression occurred because loss of ENG in endothelial cells leads to increased sensitivity to VEGF and a hyperproliferative response. Development of AVMs and associated progression to high-output heart failure in the absence of endothelial ENG was attenuated by targeting VEGF signaling with an anti-VEGFR2 (VEGF receptor 2) antibody.

**Conclusions:**

ENG promotes the normal balance of VEGF signaling in quiescent endothelial cells to maintain vessel caliber—an essential function in conditions of increased VEGF expression such as local hypoxia or inflammation. In the absence of endothelial ENG, increased sensitivity to VEGF drives abnormal endothelial proliferation in local regions of high VEGF expression, leading to AVM formation and a rapid injurious impact on heart function.

## Abstract 2

Supplemental Digital Content is available in the text.

**Meet the First Author, see p 160**

ENG (endoglin) protein (also known as CD105 [cluster of differentiation 105]) is a coreceptor for members of the TGF (transforming growth factor)-β superfamily of ligands, with high affinity for BMP (bone morphogenetic protein) 9 and 10.^1–3^ ENG is expressed in vascular endothelial cells (ECs), mesenchymal stem cells, myofibroblasts, syncytiotrophoblasts, and macrophages.^4–6^ In ECs, ENG promotes BMP 9/10 signaling through the receptor ACVRL1 (also known as ALK1 [activin receptor-like kinase 1]) to regulate developmental angiogenesis,^7^ endothelial migration, and cell shape.^8,9^ Mutations in *ENG* or *ACVRL1* lead to the inherited vascular disorder, hereditary hemorrhagic telangiectasia (HHT), a disease characterized by arteriovenous malformations (AVMs),^10^ which are direct connections between arteries and veins. We and others have shown using preclinical models that developmental or pathological angiogenesis is an essential trigger driving the vascular remodeling associated with AVM formation in the developing retina, adult brain, and dermal wound healing.^11–14^ It is also well established that ENG plays a critical role in angiogenesis, as ubiquitous loss of ENG (*Eng*^−/−^) leads to major angiogenesis defects in the developing embryo and early embryonic lethality.^15^ Nevertheless, the molecular and cellular mechanisms that drive abnormal vascular remodeling events in quiescent blood vessels to generate AVMs in adult HHT patients remain obscure. Furthermore, there is a growing interest in ENG in cardiovascular disease more broadly, as the extracellular domain of ENG known as soluble ENG is found at increased levels in the circulation (most likely due to shedding from the EC surface) in preeclampsia,^16^ hypertension,^17^ hypercholesterolemia,^18^ and in patients with increased left heart filling pressures.^19^ However, whether soluble ENG is simply a biomarker of disease or has an important biological role in regulating the mature vasculature is unknown. These studies are severely hampered by our lack of understanding of the full-length transmembrane form of ENG in quiescent mature ECs and specifically whether it plays a key role in maintaining the mature cardiovasculature, which is the focus of this investigation.

## Methods

The data that support the findings of this study are available from the corresponding author on reasonable request.

All animal experiments were performed under European legislation and approved by the Animal Ethics Committees of Newcastle University and Karolinska Institute. Mice (*Cdh5(PAC)-CreER*^*T2*^*;Eng*^*fl/fl*^) were generated as described previously,^11^ maintained in a C57BL/6 background on standard chow, and crossed with transgenic fluorescent reporter mouse lines B6.Cg-*Gt(ROSA)26Sor*^*tm3(CAG-EYFP)Hze*^/J (No. 007903; Jackson Laboratory) for in vivo lineage tracing. Male and female mice aged between 8 and 24 weeks were given tamoxifen (2 mg/day) either by intraperitoneal injection for 5 consecutive days or by gavage for 3 consecutive days to deplete ENG in ECs. Tamoxifen-treated *Eng*^*fl/fl*^ littermates were used as controls. Mice were assigned to experiments based on genotype, but identities were then anonymized so that researchers were normally blinded to genotype. To measure cell proliferation, intraperitoneal injections of 20 mg/kg of 5-ethynyl-2-deoxyuridine (EdU) were given at day 0 (first day of tamoxifen administration) and days 2, 4, 6, 8, and 10. Mice were humanely killed at day 13 and tissues analyzed using the Click-iT EdU imaging kit (C10338; ThermoFisher Scientific). All image processing and analysis was performed using ImageJ software. Antibody DC101 was a kind gift from Eli Lilly and delivered intraperitoneally at 40 mg/kg as described in the main text. Western blot, quantitative polymerase chain reaction, tissue analyses, and cardiac magnetic resonance imaging were performed as described previously^6,11,20–22^ with further details and data analysis methods available in Online Data Supplement.

## Results

**Figure 1.**
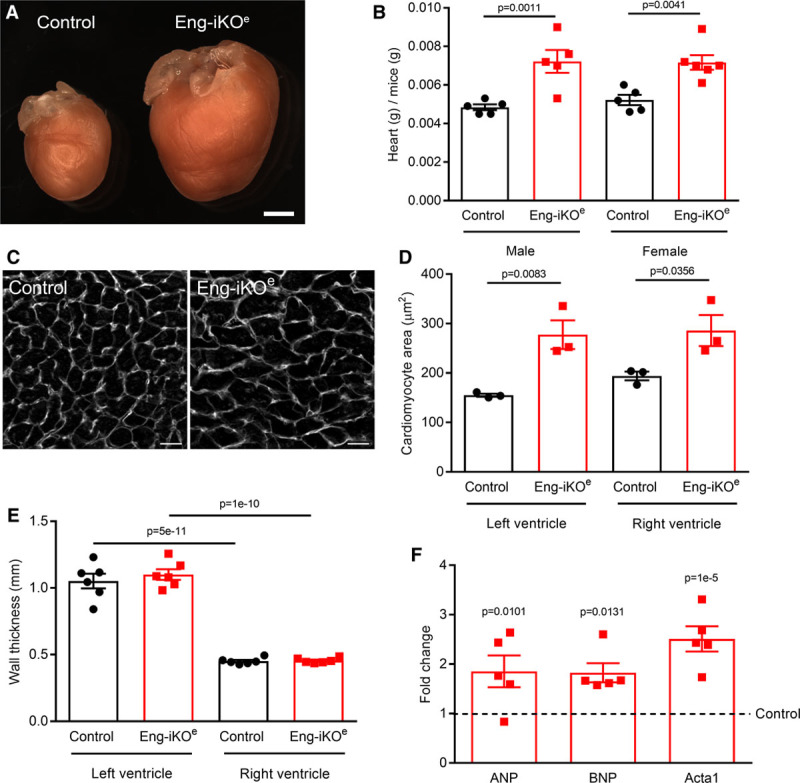
**Loss of endothelial ENG (endoglin) leads to high cardiac output and cardiac hypertrophy associated with expression of heart failure markers**. **A**, Eng-iKO^e^ mice show a progressive heart enlargement. This example is 8 wk after endothelial ENG depletion. Scale bar=2 mm. **B**, Hearts from Eng-iKO^e^ mice are significantly larger and heavier than controls 5 wk after ENG depletion (n≥5/group). **C** and **D**, Heart sections were stained with fluorescein-conjugated wheat germ agglutinin to outline individual cardiomyocytes, which were significantly larger 5 wk after ENG depletion compared with controls (n=3/group). Scale bar=20 µm. **E**, Average wall thickness of end diastolic left and right free wall measured by cardiac magnetic resonance imaging 5 wk after ENG depletion (n=6/group). **F**, Quantitative polymerase chain reaction shows increased levels of *Anp*, *Bnp*, and *Acta1* in Eng-iKO^e^ heart tissue 5 wk after ENG depletion (n=5/group). All data were analyzed by 2-way ANOVA with Bonferroni correction.

To investigate the role of endothelial ENG in established mature cardiovasculature, we genetically depleted *ENG* in adult *Cdh5(PAC)-CreER*^*T2*^*;Eng*^*fl/fl*^ mice using transient tamoxifen treatment to generate endothelial-specific *Eng* knockout mice (Eng-iKO^e^). Loss of endothelial ENG protein in tissues known to be affected in HHT (lung, liver, brain, and gut) was confirmed by immunofluorescent staining (Online Figure IA). The effect of ENG depletion from vascular endothelium was striking; Eng-iKO^e^ mice rapidly developed a grossly enlarged heart and cardiomyocyte hypertrophy (Figure 1A through 1D), while ventricular wall thickness was unchanged, consistent with eccentric cardiac remodeling (Figure 1E). Myocardial tissue also showed upregulation of *ANP* (*Nppa*), *BNP* (*Nppb*), and *Acta1* genes, associated with progression to cardiac failure (Figure 1F), but congestive heart failure appeared unlikely because there was no accompanying change in peripheral oxygen saturation, heart rate, or tissue edema (Online Figure II).

**Figure 2.**
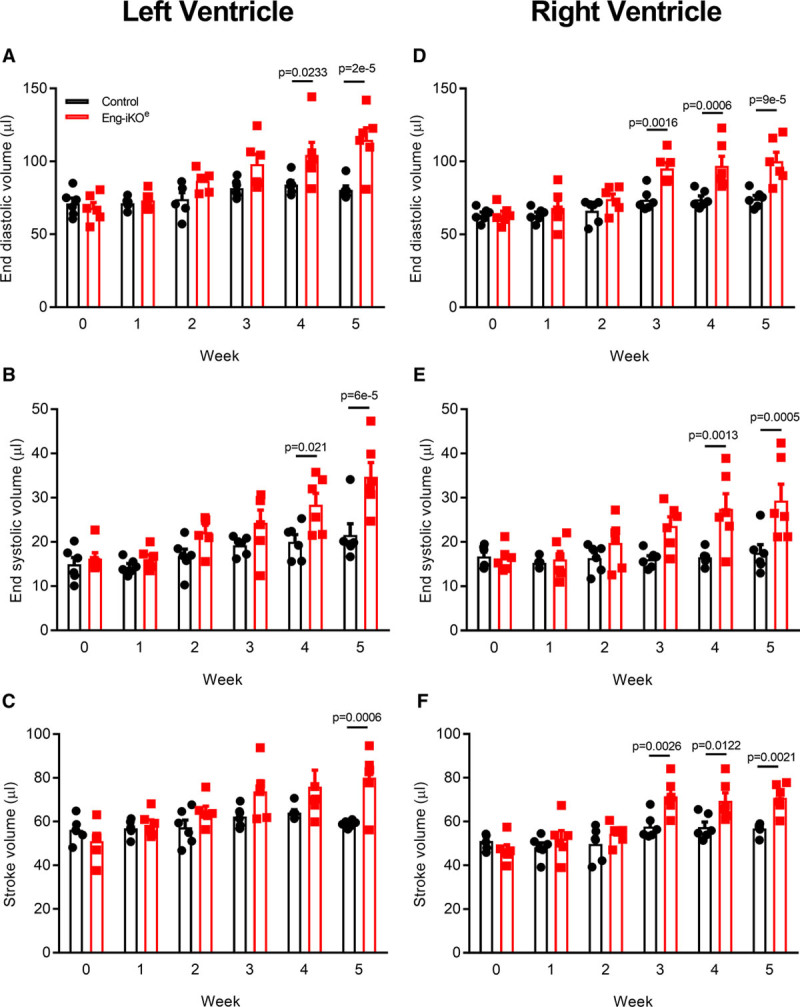
**Loss of endothelial ENG (endoglin) leads to increased end systolic and end diastolic volumes and increased stroke volumes in left and right ventricles**. Longitudinal study of heart function in control and Eng-iKO^e^ mice using cardiac magnetic resonance imaging demonstrates a progressive increase in right and left ventricular volumes over 5 wk following endothelial ENG depletion (**A**–**C**) compared with control mice (**D**–**F**). Significant changes are observed in the right ventricle within 3 wk of endothelial ENG loss, while similar changes occur in the left ventricle by 4 wk (n=6/group). All data were analyzed by 2-way ANOVA with Bonferroni correction.

Cardiac magnetic resonance imaging was performed in a longitudinal 5-week study to evaluate the impact of endothelial ENG depletion on heart function and revealed a progressive increase in end diastolic and end systolic volumes in both left and right ventricles, associated with increased stroke volumes. These changes occurred initially in the right ventricle but were subsequently mirrored in the left ventricle (Figure 2) and accompanied by increased left ventricular mass and increased cardiac output (Online Table I). Follow-up analyses showed that ventricular volumes further increased over 12 weeks, accompanied by a significant drop in ejection fraction (Online Figure IIIA). This phenotype is characteristic of high-output heart failure (HOHF). Disease progression occurred in a highly reproducible way affecting 100% male and female Eng-iKO^e^ mice and was followed by significant weight loss and >70% mortality at 24 weeks after endothelial ENG knockdown (Online Figure IIIC and IIID). To investigate the primary effects resulting from loss of endothelial ENG, we performed subsequent assessment of the cardiovascular phenotype no later than 5 weeks after ENG depletion, before any clinical signs of ill health.

**Figure 3.**
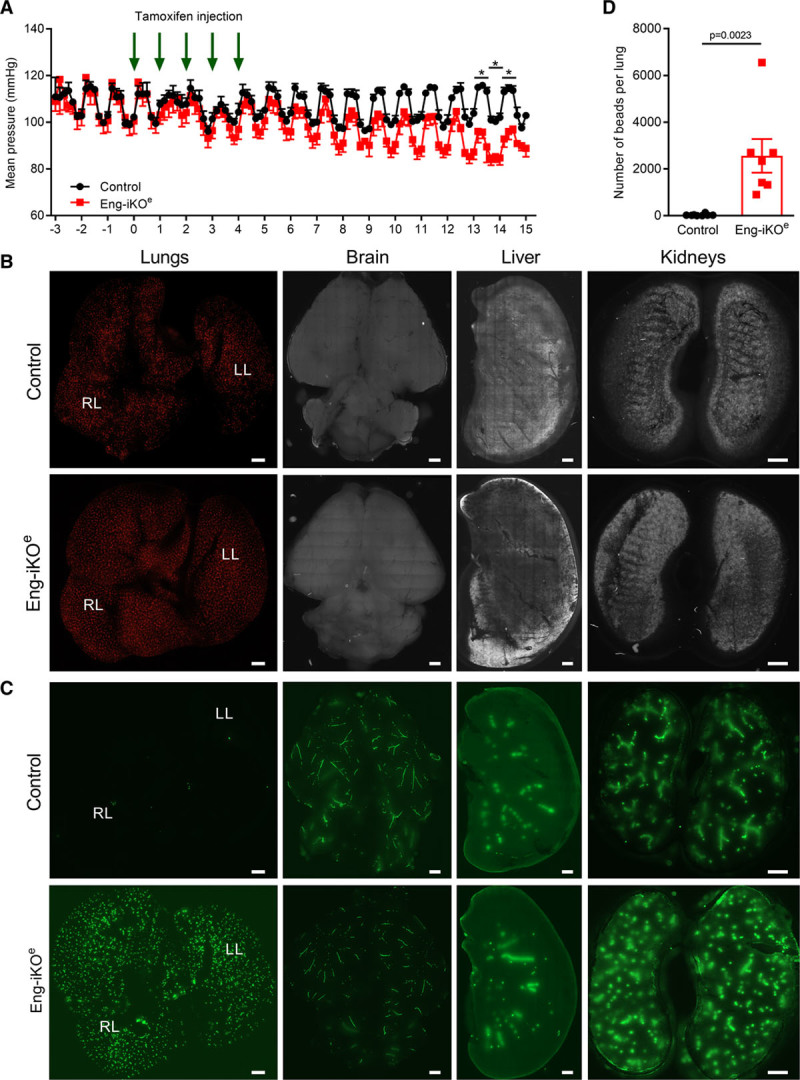
**Rapid loss of aortic blood pressure (BP) and extensive left-to-right shunting from the systemic to the pulmonary vasculature in Eng-iKO^e^ mice**. **A**, Aortic telemetry shows the diurnal rhythm of BP in both groups of mice, with higher BP during the night when mice are the most active and lower BP during the day when they are asleep. Baseline measurements were made for 3 d before tamoxifen was delivered once daily for 5 consecutive days, and mean aortic BP subsequently falls in Eng-iKO^e^ mice (n=4/group). Data analyzed by 2-way ANOVA with Bonferroni correction. *P<0.05, with the full set of *P* in Online Table V. **B**, Small fluorescent microbeads (15 µm^3^) injected into the tail vein of Eng-iKO^e^ and control mice are all efficiently trapped in the lung vasculature, indicating absence of pulmonary arteriovenous malformations. No beads were detected in brain, kidney, or liver (n≥6/group). **C**, Large fluorescent microbeads (45 µm^3^) injected into the left ventricle of control mice are efficiently trapped in the vasculature of the systemic organs (brain, kidney, and liver) with few beads reaching the lungs. However, in Eng-iKO^e^ mice, the microbeads rapidly reach the lungs within 1 min of bead delivery to the left ventricle (n≥8/group). Scale bar=2 mm. **D**, Quantification of microbeads used in **C**, reaching lungs of Eng-iKO^e^ and control mice, analyzed by Mann-Whitney *U* test (n≥7/group). LL indicates left lung; and RL, right lung.

Clinically, HOHF can result from loss of vascular resistance. Aortic telemetry was, therefore, used to investigate real-time changes in aortic blood pressure (BP) in response to loss of endothelial ENG. Daily aortic BP cycles in parallel with mouse activity, being the highest at night. However, ENG knockdown led to a significant reduction in mean aortic BP, demonstrating loss of peripheral vascular resistance compared with baseline (Figure 3A). Consistent with this finding, cardiac-conductance catheter measurements showed reduced end systolic left ventricular BP in Eng-iKO^e^ mice at 1 week after endothelial ENG knockdown compared with controls (Online Table II). This was accompanied by an increased right ventricular contractility (Max dp/dt) and increased right ventricular end diastolic pressure indicating an increase in cardiac preload in Eng-iKO^e^ mice (Online Table II). This phenotype is consistent with a major loss of systemic vascular resistance associated with increased venous return and greater right ventricular filling pressure. These data suggest that increased preload drives the observed eccentric cardiac enlargement, as predicted by the Frank-Starling law, resulting in high cardiac output.

As reduction in systemic aortic BP may arise as a consequence of anemia or arteriovenous shunting, the phenotype of Eng-iKO^e^ mice was investigated accordingly. There was no sign of hemorrhage in Eng-iKO^e^ mice at 1 month after ENG depletion, and blood analysis showed normal hematocrit and hemoglobin levels, excluding anemia as a potential cause of HOHF (Online Table III). Because HHT1 (the form of HHT attributable to mutations in *ENG*) is associated with development of pulmonary AVMs,^23^ we next evaluated pulmonary arteriovenous shunting between venous and arterial beds in Eng-iKO^e^ mice. Fluorescent microspheres (15 µm^3^), which are too large to pass through normal murine pulmonary capillaries, were injected into the tail vein 1 month after ENG knockdown. All beads were found efficiently trapped in pulmonary vasculature of both control and Eng-iKO^e^ mice (Figure 3B), with no beads detected in systemic organs (brain, kidney, and liver) consistent with the absence of pulmonary AVMs and also in line with the increased right ventricular pressure that we observed previously (Online Table II). In addition, blood taken from Eng-iKO^e^ mice showed normal oxygen saturation compared with controls (Online Table III), while oximetry confirmed similar peripheral oxygen saturation in control and Eng-iKO^e^ mice (Online Figure II). All these data are in agreement with the conclusion that there are no pulmonary AVMs in Eng-iKO^e^ mice.

**Figure 4.**
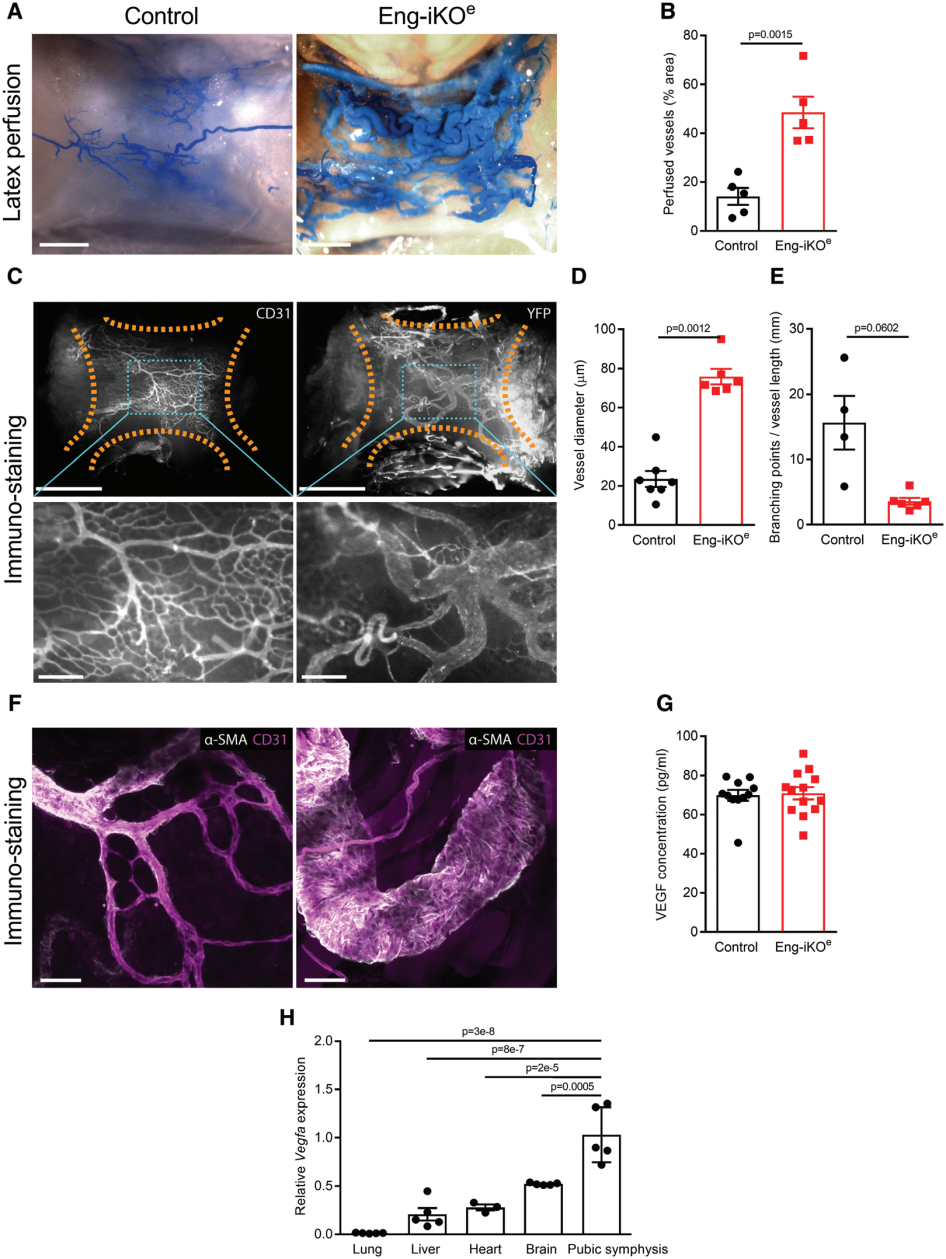
**Rapid development of arteriovenous malformations (AVMs) in association with pubic symphysis following endothelial cell–specific ENG (endoglin) deletion in adult mice**. **A**, Ventral vessels of the pubic symphysis in control (**left**) and Eng-iKO^e^ (**right**) mice, following systemic perfusion with latex (blue). Scale bar=1 mm. **B**, Area occupied by latex perfused vessels as a proportion of the total pubic symphysis area (marked by dotted lines) is significantly greater in Eng-iKO^e^ mice compared with controls. Data were analyzed by Student unpaired *t* test (n=5/group). **C**, Pubic symphysis of control or Eng-iKO^e^ mice, stained for CD31 (cluster of differentiation 31) or YFP (yellow fluorescent protein) to visualize the vasculature. Upper panels (scale bar=1 mm). Bottom panels (scale bar=200 μm) correspond to boxed areas of upper panels (scale bar=1 mm). **D**, Quantification of vessel diameters (vessel area/vessel length, averaged across individual vessels, excluding capillaries with a diameter <8 μm) of control (n=7) and Eng-iKO^e^ (n=6) mice reveals increased vessel size following ENG deletion. Data analyzed by Mann-Whitney *U* test. **E**, Trend toward reduced number of branch points following ENG deletion (n=4–6 mice). Data were analyzed by Student unpaired *t* test with Welch correction. **F**, Staining for αSMA (alpha smooth muscle actin; white) and CD31 (cluster of differentiation 31; magenta) reveals extensive coverage of AVMs by αSMA-positive cells. Scale bar=50 µm. **G**, No difference in circulating VEGF (vascular endothelial growth factor) protein was observed between control and Eng-iKO^e^ mice 5 wk after ENG depletion. Data were analyzed by Mann-Whitney *U* test (n=11–13/group). **H**, Expression of *Vegfa* transcripts assessed by quantitative polymerase chain reaction is significantly higher in pubic symphysis cartilage compared with other tissues in normal control mice (n=3–5/group). Data were analyzed by 1-way ANOVA with Bonferroni correction.

HOHF is a rare complication of HHT mainly suggested to be a consequence of arteriovenous shunting in the liver.^24^ Although hepatic AVMs are more frequent in HHT2 (ALK1 patients) than HHT1 (ENG patients) they could potentially develop after endothelial ENG depletion and contribute to the observed reduction in aortic BP. To evaluate this possibility, microbeads were delivered to the systemic circulation via the left cardiac ventricle. Larger 45-µm^3^ beads were required for this analysis because 15-µm^3^ beads readily passed through the hepatic vasculature from the arterial to the venous side in control mice (not shown), consistent with the large sinusoidal capillaries and the presence of natural arterioportal venular anastomoses in normal rodent liver.^25^ These large beads were efficiently trapped in the systemic vasculature in control mice but readily crossed to the lung following endothelial ENG depletion leading to a massive accumulation of beads in lungs of Eng-iKO^e^ mice (Figure 3C and 3D). This pointed to de novo arteriovenous shunting at ≥1 sites in the systemic vasculature. To map any changes in liver vessel architecture, vascular tracing was performed using radio-opaque microfil and tissues analyzed by micro-CT. However, no vascular malformations were seen in livers of Eng-iKO^e^ mice 1 month after ENG depletion (Online Figure IVA). In parallel, vascular tracing of the skin, gastrointestinal tract, spleen, and kidney was performed using latex. This compound is reportedly too large to pass from arteries to veins in mice and has been widely used to visualize arteriovenous connections. However, we observed that latex perfusion data need to be interpreted with caution as latex readily passes from the distal tail artery to tail veins and from distal leg arteries to femoral veins, reaching the vena cava in 80% (12 of 15) control mice and on occasion filling smaller veins in a retrograde manner. Therefore, only sites of direct antegrade flow of latex from arteries into veins were recorded to ensure that AVMs were correctly identified. We observed that latex was retained within arterial vessels of skin, gastrointestinal tract, spleen, and kidney vasculature and rarely transferred to veins of these organs in either control or Eng-iKO^e^ mice, consistent with the general absence of AVMs following loss of endothelial ENG (Online Table IV and Online Figure IVB). Strikingly, however, AVMs were consistently and reproducibly observed in vessels associated with the cartilage of the pubic symphysis within 1 week of ENG depletion in both male and female mice (Figure 4A and 4B; Online Table IV). These vessels were over 3× increased in diameter compared with control vessels in the same region (Figure 4C and 4D). There was no loss of vascular smooth muscle, which appeared to extensively coat these enlarged vessels (Figure 4F).

**Figure 5.**
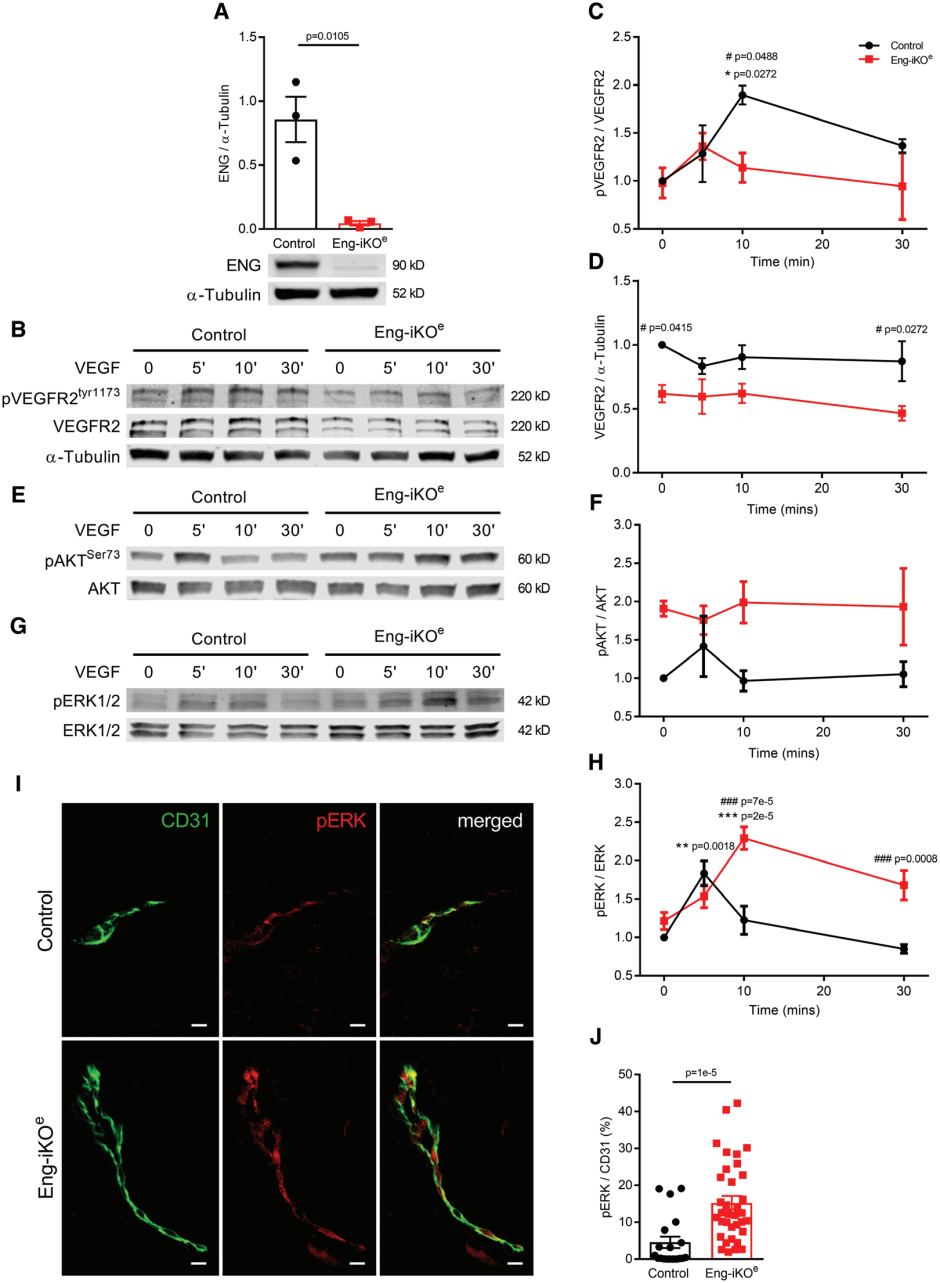
**Endothelial cells (ECs) show altered VEGF (vascular endothelial growth factor) signaling responses following loss of ENG (endoglin)**. **A**, ECs cultured from *Rosa26-Cre*^*ERT2*^*;Eng*^*fl/fl*^ mice were transiently exposed to 4OH-tamoxifen to deplete ENG protein, which is measured by Western blot. Data were analyzed by Student unpaired *t* test (n=3). **B**–**D**, Cells exposed to VEGF (10 ng/mL) for 0, 5, 10, and 30 min and assessed by Western blot show a reduced VEGFR2 (VEGF receptor 2) phosphorylation response and reduced overall VEGFR2 levels compared with control ECs. **E** and **F**, Basal pAKT (phospho-protein kinase B) activity appears enhanced in Eng-iKO ECs compared with controls. **G** and **H**, Eng-iKO ECs have an increased and prolonged pERK (phospho-extracellular signal-regulated kinase) response compared with control cells. Molecular weights of named proteins are predicted based on molecular weight standards. Data in **C**, **D**, **F**, and **H** were analyzed using 2-way ANOVA with Bonferroni correction. Asterisks (*) indicate significant changes with respect to baseline for each genotype, while hashes (#) indicate significant differences between control and Eng-iKO ECs; the full set of *P* is summarized in Online Table VI (n=3–5/group). **I**, Increased levels of pERK in ECs of pubic symphysis of Eng-iKO^e^ compared with control mice (scale bar=10 µm). **J**, Quantification of pERK vascular coverage from individual vessels from control (6 mice, n=19 vessels) and Eng-iKO^e^ (4 mice, n=35 vessels) mice. Data analyzed by Mann-Whitney *U* test. AKT indicates protein kinase B; CD31, cluster of differentiation 31; and ERK, extracellular signal-regulated kinase.

**Figure 6.**
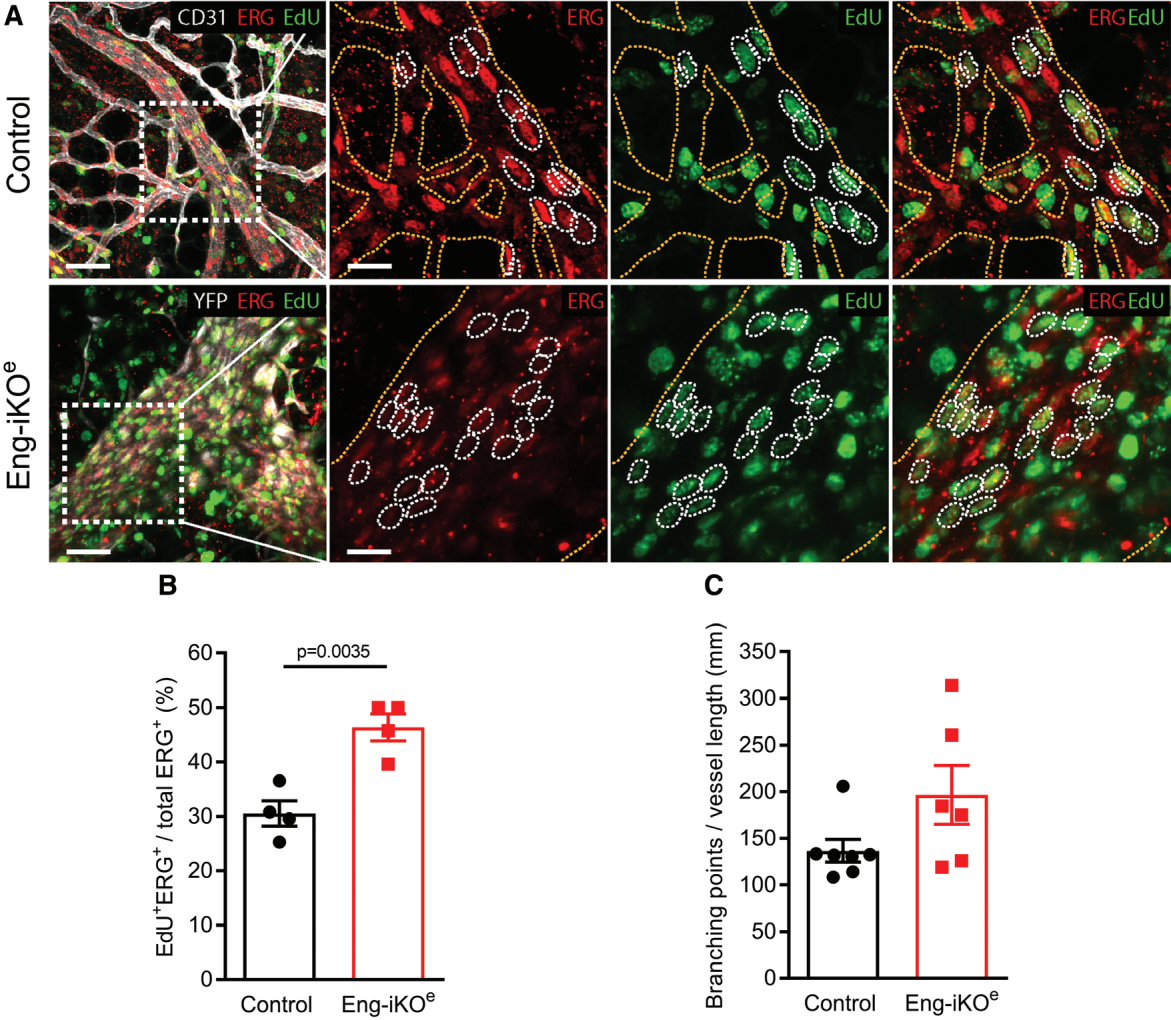
**Endothelial cell (EC) proliferation of the adult vasculature is promoted by endoglin deletion**. **A**, Vessels of the pubic symphysis of control (**top**) and Eng-iKO^e^ (**bottom**) mice, exposed to repeated 5-ethynyl-2-deoxyuridine (EdU) injections over 2 wk, stained for CD31 (cluster of differentiation 31) or YFP (yellow fluorescent protein; ECs, white), ERG (erythroblast transformation-specific transcription factor; EC nuclei, red), and EdU (proliferating cells, green). **Left**, Merged overviews. Scale bars=50 µm. **Right**, Boxed areas in **left**. Scale bars=20 µm. Yellow dotted lines outline the vasculature, and white dotted lines indicate proliferating ECs (ERG^+^ and EdU^+^, double positive). **B**, Quantification of accumulated EC proliferation over 2 wk demonstrates an increase from 31% proliferating ECs in control (n=4 mice) to 45% in Eng-iKO^e^ (n=4 mice). Data were analyzed by Student unpaired *t* test. **C**, Quantification of average EC size calculated from the number of ERG+ cells per vessel shows a trend toward an increased EC size in Eng-iKO^e^ mice (n=6) compared with controls (n=7). Data were analyzed by Mann-Whitney *U* test.

**Figure 7.**
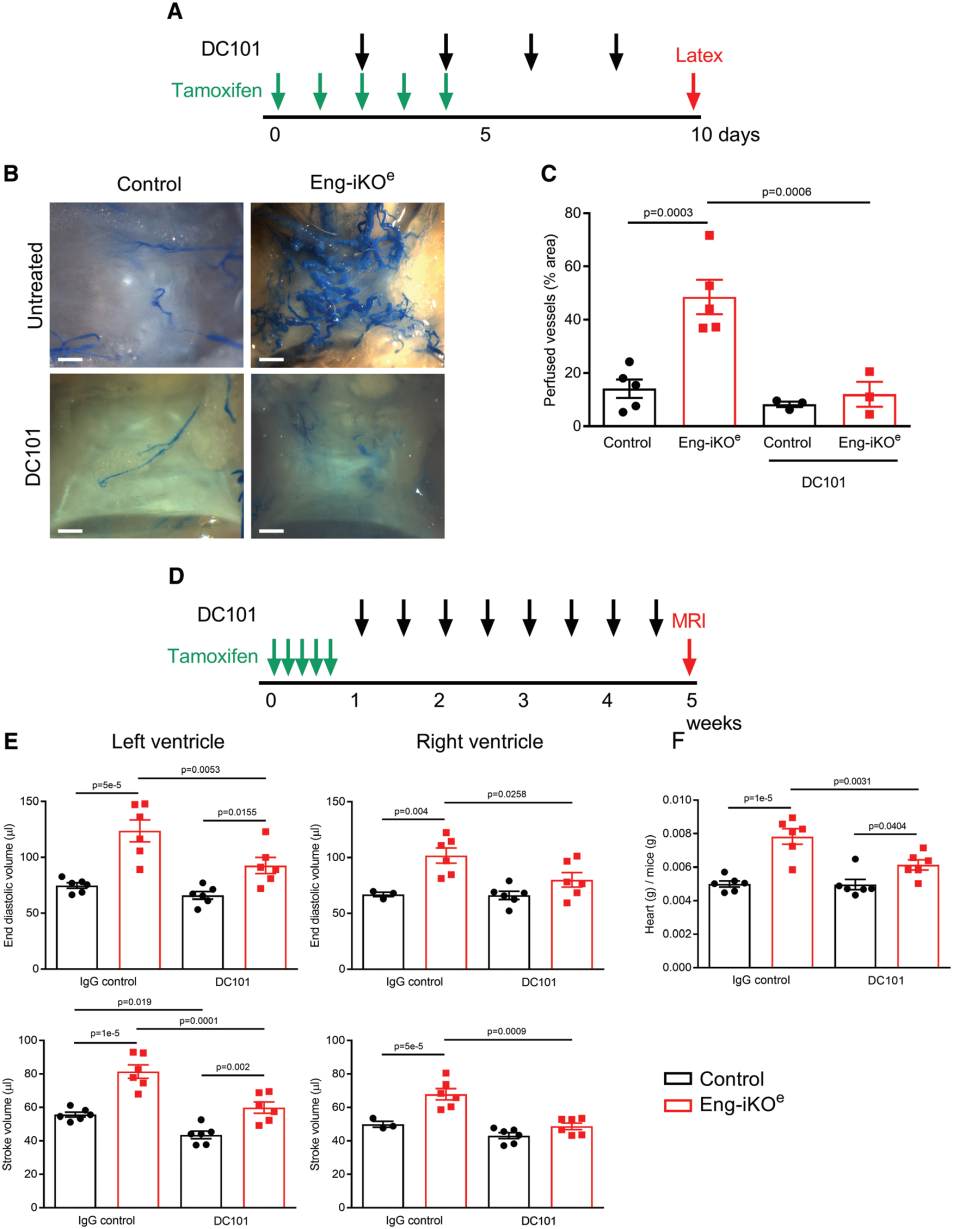
**Anti-VEGFR2 (VEGF receptor 2) therapy protects against arteriovenous malformations (AVMs) and high-output heart failure in Eng-iKOe mice**. **A**, Adult control and Eng-iKO^e^ mice were treated with tamoxifen (2 mg/d for 5 d) and DC101 every other day for 8 d. **B**, Tissue was harvested following latex perfusion at day 10, and DC101 treatment completely abrogated AVM formation in the pelvic region of Eng-iKO^e^ mice. Scale bar=0.8 mm. **C**, Quantitation of perfused vessels in pelvic area (n=3–5/group). **D**, Adult mice were treated with tamoxifen (2 mg/d for 5 d), and DC101 (or a nonspecific IgG) was given twice weekly from day 8 for 5 wk. **E**, Cardiac magnetic resonance imaging (MRI) analysis revealed that DC101 treatment improved cardiac function in Eng-iKO^e^ mice and the detrimental cardiac remodeling parameters (end systolic volume, end diastolic volume, and stroke volume) were all significantly protected by DC101 treatment (n=3–6/group). **F**, Heart mass of Eng-iKO^e^ mice was significantly reduced by DC101 treatment (n=6/group). All data were analyzed by 2-way ANOVA with Bonferroni correction.

**Figure 8.**
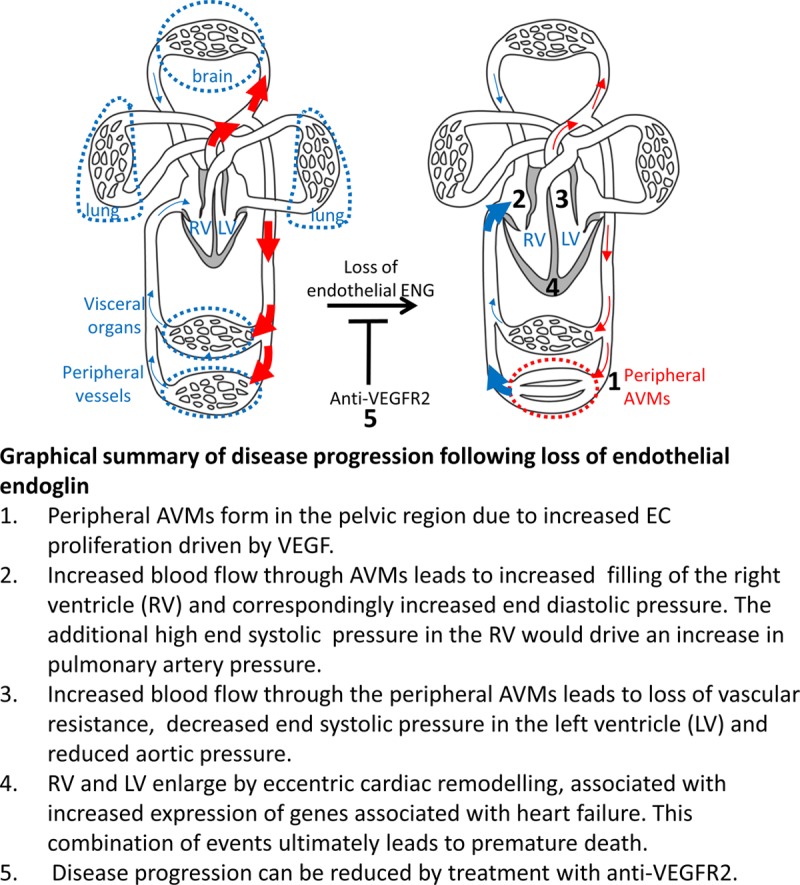
**Schematic illustration of events leading to heart failure following endoglin depletion in endothelial cells**. AVM indicates arteriovenous malformation; ENG, endoglin; LV, left ventricle; RV, right ventricle; VEGF, vascular endothelial growth factor; and VEGFR2, VEGF receptor2.

To investigate possible factors driving the formation of these AVMs, we first confirmed that baseline ENG levels in the pubic symphysis are similar to other tissues (Online Figure IC). We then looked at circulating VEGF (vascular endothelial growth factor) concentrations, as these have previously been reported to be increased in HHT patients.^26^ However, circulating VEGF levels were similar in Eng-iKO^e^ and control mice (Figure 4G) consistent with the idea that no systemic proangiogenic stimulus was present in Eng-iKO^e^ adult mice. We next considered there might be a local angiogenic stimulus in the vicinity of the pubic symphysis. We found this cartilage to have no difference in TGFβ1 expression between control and Eng-iKO^e^ (Online Figure ID), but it had significantly increased VEGF levels compared with other organs (liver, heart, brain, and lung) in line with the known hypoxic properties of cartilage tissue^27^ (Figure 4H). However, this is an unusual region of fibrocartilage that is unprotected by a synovial capsule and has a highly intimate association with the feeding vasculature (Online Figure VA).^28^ This contrasts to joint cartilage where blood vessels are adjacent to but not intimately embedded within the cartilage (Online Figure VB). Therefore, blood vessels within fibrocartilage of the pubic symphysis would normally be exposed to increased levels of VEGF and require tight regulation of the VEGF response. As ENG is a coreceptor for BMP9—a circulating vascular quiescent factor that antagonizes the proangiogenic effects of VEGF^29,30^—we questioned whether exaggerated endothelial responses to VEGF signaling occurred in the absence of ENG and could be driving de novo vascular remodeling in mature adult vasculature of the pelvic region. To investigate EC autonomous defects, ECs were cultured from *Rosa26-Cre*^*ERT2*^*;Eng*^*fl/fl*^ mice as described previously.^31,32^ Transient treatment with 4-hydroxytamoxifen was used to deplete ENG (Figure 5A). ENG-depleted ECs showed reduced basal expression of VEGFR2 (VEGF receptor 2) and reduced VEGFR2 phosphorylation in response to VEGF stimulation (Figure 5B through 5D), in agreement with a previous report.^33^ Eng-iKO^e^ ECs also appear to have increased basal pAKT (phospho-protein kinase B) levels (Figure 5E and 5F) that may promote their increased survival. However, most striking was an amplified pERK (phospho-extracellular signal-regulated kinase) response to VEGF stimulation in Eng-iKO^e^ ECs (Figure 5G and 5H). This was also confirmed in vivo with significantly increased pERK staining in ECs of Eng-iKO^e^ pubic symphysis compared with controls (Figure 5I and 5J). As phosphorylation of ERK (extracellular signal-regulated kinase) is associated with increased cell proliferation, we evaluated this property and found there was a significant increase in EC proliferation in AVMs of the pubic symphysis of Eng-iKO^e^ mice (Figure 6A and 6B) that was not seen in brain ECs (Online Figure VI). There was also a trend toward increased EC size in vessels of the pubic symphysis. Taking this together, we concluded that loss of endothelial ENG resulted in increased sensitivity to VEGF that led to AVMs promoted by increased EC proliferation. In light of this finding, we reasoned that inhibition of VEGF signaling should reduce the incidence and severity of AVMs in Eng-iKO^e^ mice and improve cardiac outcomes. In line with this idea, we found that short-term treatment with anti-VEGFR2 antibody DC101 (40 mg/kg) given to mice after ENG knockdown completely prevented AVM formation (Figure 7A through 7C). In addition, longer term studies using a similar DC101 treatment ameliorated the abnormal HOHF cardiac phenotype of Eng-iKO^e^ mice. Cardiac stroke volumes and adverse remodeling of both cardiac ventricles and cardiac hypertrophy were all reduced in DC101-treated Eng-iKO^e^ mice compared with those treated with nonspecific IgG (Figure 7D through 7F). Our data, therefore, support the concept that ENG is required to maintain endothelial quiescence in adult life where it acts to counter an overexuberant endothelial proliferation response to VEGF signaling, thereby protecting the vasculature against development of AVMs. Furthermore, rapid development of peripheral AVMs in the absence of endothelial ENG has a major effect on the heart leading to a rapid drop in aortic BP, increased venous return to the heart, and an increased cardiac output that invariably progresses to HOHF (Figure 8).

## Discussion

We show for the first time that endothelial ENG is essential in normal adult life to maintain vascular architecture and cardiac function. We demonstrate rapid development of large pelvic AVMs following loss of ENG in adult ECs exposed to endogenous VEGF in the noncapsulated fibrocartilage of the pelvic symphysis. These enlarging AVMs lead to loss of aortic BP and increased cardiac filling pressure, which drives stretching of the myocardium during diastole and increased stroke volume in accordance with the Frank-Starling law. The resultant progressive ventricular enlargement and high cardiac output ultimately leads to reduced ejection fraction and heart failure. These effects on the heart are similar to those seen in patients given surgical arteriovenous fistulas to initiate kidney dialysis.^34^

A small number of HHT patients develop HOHF because of left-to-right shunting of blood through major hepatic AVMs, but these are rare and more frequently seen in ALK1 (HHT2) patients than in ENG (HHT1) patients, for reasons that are not yet understood.^23^ In agreement with the low clinical prevalence in HHT1, there was no evidence of hepatic AVMs following loss of endothelial ENG in our model. We observe a novel pelvic anatomic location for AVMs that may also be highly relevant in humans with HHT; there have been limited reports of pelvic AVMs in HHT patients,^35^ but pelvic AVMs are not yet screened for in HHT. Although challenging to diagnose due to their deep location, pelvic AVMs are important to evaluate in patients as they carry a high risk for developing HOHF.^36–38^

Furthermore, as HHT1 patients carry one wild-type copy of the *ENG* gene, the current premise is that a second hit (somatic loss of function mutation in normal *ENG* allele) is required to generate a vascular location that is at risk for detrimental remodeling to generate AVMs.^13,39^ In addition, the vascular lesions in HHT patients generally develop in tissues exposed to environmental or inflammatory insults (lung, skin, and gastrointestinal tract) and liver (responsible for removing toxins of environmental origin). This complex pathogenesis explains the variability of clinical symptoms even within the same HHT1 family carrying the same *ENG* mutation. In other words, the exact site and timing of lesions is unpredictable because of the timing of the second hit (random stochastic mutation event) and the subsequent exposure to an inflammatory or other environmental stimulus that promotes angiogenesis; for example, macrophages secrete abundant VEGF protein. Therefore, although our model does not recapitulate the usual tissue specificity of HHT, the factors driving the formation of AVMs are the same. In the mouse model, loss of ENG occurs after tamoxifen treatment, and the environmental proangiogenic trigger is the local high level of VEGF within the fibrocartilage. Therefore, the clinical significance of our findings is that HHT1 patients with a second genetic hit in the *ENG* gene in any tissue exposed to high levels of VEGF will develop AVMs.

VEGF is an important protein produced in the naturally hypoxic cartilage tissue, where it acts as a survival factor for chondrocytes.^40,41^ Most cartilage is enclosed within a synovial capsule, but the pubic symphysis is an unusual fibrocartilage with intimate association of the vasculature and cartilage tissue.^28^ The evidence we show here links the naturally high endogenous VEGF levels in the exposed cartilage of the pubic symphysis, combined with the altered VEGF response of ENG-deficient ECs, to drive increased EC proliferation leading to large AVMs in the pelvic region and consequent HOHF. Furthermore, we show for the first time that treatment with DC101 to target VEGFR2 effectively protects against development of AVMs and reduces the detrimental effects of ENG loss on heart function.

A degree of serendipity led to the realization that targeting VEGF signaling had potential value in treating HHT patients. Anti-VEGFA therapy (bevacizumab) originally given to HHT patients for concurrent cancer led to a marked improvement of HHT disease symptoms^42,43^ and was followed up recently in a successful phase 2 clinical trial in a small number of HHT2 patients with HOHF.^44^ However, there is little understanding of the molecular interaction of VEGF signaling and disrupted BMP9 signaling in HHT, which is essential to optimize and improve these therapies. We have recently shown that reduced VEGFR1 levels exacerbate HHT symptoms,^45^ in line with the current concept that VEGFR1 normally acts as a ligand sink to divert VEGFA signaling away from VEGFR2. We now provide a valuable model to test and optimize therapeutic targeting of the VEGF signaling pathway in HHT disease.

Our previous work suggested altered VEGFR2 membrane recycling kinetics associated with ENG-depleted ECs.^8^ Here, we additionally observe reduced VEGFR2 receptor levels in ENG-deficient ECs similar to a previous report^33^ and also observed in SMAD4 (mothers against decapentaplegic homolog 4) deficient ECs in vivo in a model that develops AVMs in the neonatal retina.^46^ Downregulation of VEGFR2 expression in ECs may reflect an adaptation to the increased sensitivity to VEGF signaling in the absence of ENG. We show here for the first time that ENG-depleted ECs in vitro show an exaggerated pERK response to VEGF compared with controls, and this increased pERK response was confirmed in the enlarged vessels of the Eng-iKO^e^ pubic symphysis in vivo. Increased pERK activity likely drives the increased EC proliferation associated with AVM formation. Intriguingly increased ERK activity in ECs is also associated with spontaneous brain AVMs because of somatic activating mutations in KRAS (Kirsten rat sarcoma viral oncogene homolog)^47^ and may represent a unifying mechanism for AVM pathogenesis.

ENG is required to promote BMP9 signaling in the context of blood flow,^48^ and BMP9 circulates in an active form in vivo,^49^ although more recent data suggest its active form is a BMP 9/10 complex.^50^ Our data suggest that ENG-mediated BMP 9/10 signaling counters the VEGF response to reduce pERK responses and protect the vasculature. A recent report showed that a minimum of 2-hour preincubation of cells with BMP9 was required to block VEGF-stimulated cell responses,^51^ suggesting there are yet unidentified downstream factors responsible for ENG/ALK1/BMP9 regulation of the EC responses to VEGF.

Recent work revealed PI3K (phosphoinositide 3-kinase) signaling defects in response to VEGF in the neonatal retina when ALK1 levels are reduced,^51,52^ but targeting VEGFR2 did not protect against AVMs.^52^ Similarly, targeting VEGFR2 in the Eng-iKO^e^ neonatal model did not significantly reduce the number of arteriovenous shunts in the neonatal retina but did reduce their diameter.^8^ However, disrupting VEGF signaling interrupts the entire programme of vessel development in the neonatal retina and highlights the advantage of using an adult model to evaluate protective therapies in adult HHT patients. Furthermore, we and others have shown that endothelial-specific, or total, depletion of ALK1 in adult life leads to caecal bleeding, anemia, and rapid mortality.^21,53,54^ Pelvic AVMs were not reported in these studies but may have been present, although challenging to dissect the relative contribution of anemia and AVMs on heart pathophysiology of Alk1-iKO mice, while their rapid mortality limits longitudinal studies of disease progression and therapeutic interventions.

There is a growing interest in using anti-ENG (TRC105) antibody therapy to treat patients with cancer,^55^ but this treatment leads to development of clinical symptoms typical of HHT such as telangiectases.^56^ We suggest that patients on anti-CD105 therapy are also at increased risk of developing HOHF, and, therefore, heart function and AVM screening will be critical to ensure cardiovascular function is protected in these patients.

The model described here shows 100% penetrance of AVM development at a reproducible site in a reproducible time frame in adult mice. This represents a powerful new adult model of HHT to investigate cellular and molecular changes that underlie AVM formation and offers novel opportunities for drug testing.

The Eng-iKO^e^ mouse also provides a powerful research tool to investigate the effect of volume overload on the heart and the responsive signals involved during cardiac remodeling and hypertrophy in a timed progressive manner. Previous models of HOHF require a major surgical procedure to create a direct connection between the abdominal aorta and inferior vena cava.^57^ In contrast, our model is achieved by minimal intervention with tamoxifen to generate rapid cardiac remodeling and constitutes a valuable alternative for studies on progressive eccentric cardiac remodeling and high cardiac output associated with a range of clinical conditions including AVMs, liver disease, and morbid obesity.^58^ The data furthermore highlight the clinical importance of regional vascular abnormalities, in previously understudied regions, on heart pathology. Our study demonstrates for the first time that fluctuations of local endogenous VEGF levels can directly dictate the sensitivity to regional loss of ENG and subsequent impact on vascular integrity. This in turn strongly supports the hypothesis that ENG-dependent AVMs in adult life are prone to develop in areas of high local VEGF expression, known to occur in infection, wounding, and in hypoxic conditions, and does not depend on a systemic increase in circulating VEGF. This new and highly reproducible model will also provide an invaluable resource that will be used for further investigating AVM pathobiology in HHT and evaluating drug treatments that prevent or rescue AVMs in adult life.

## Acknowledgments

We acknowledge Electron Microscopy Research Services for image processing and thank Andrew Blamire for advice with establishing magnetic resonance imaging protocols.

## Author Contribution

H.M. Arthur, L. Jakobsson, and S. Tual-Chalot designed research study; S. Tual-Chalot, R.E. Redgrave, E. Singh, A. Lawrie, S. Luli, M. Garcia-Collado, H. Lin, Y. Wang, Y. Jin, B. Davison, and C. Park conducted experiments and acquired data; H.M. Arthur, L. Jakobsson, S. Tual-Chalot, R.E. Redgrave, E. Singh, A. Lawrie, M. Garcia-Collado, Y. Wang, Y. Jin, and C. Park analyzed data; and H.M. Arthur, S. Tual-Chalot, and L. Jakobsson prepared the manuscript.

## Sources of Funding

H.M. Arthur, B. Davison, S. Tual-Chalot, E. Singh, R.E. Redgrave, and C. Park were funded by the British Heart Foundation (PG14/86/31177, RG/12/2/29416, FS/09/027/27871, and PG/18/25/33587) and a Young Scholar Research Grant from Cure HHT (S. Tual-Chalot). L. Jakobsson was supported by the Swedish Society of Medicine, the Swedish Research Council, the Swedish Cancer Society, the Cardiovascular Programme at Karolinska Institutet, Jeanssons Stiftelser, Magnus Bergvalls Stiftelse, and Petrus och Augusta Hedlunds stiftelse. A. Lawrie was funded by British Heart Foundation Senior Basic Science Research Fellowships (FS/13/48/30453 and FS/18/52/33808).

## Disclosures

None.

## Supplementary Material

## Novelty and Significance

### What Is Known?

- Endoglin is a coreceptor in endothelial cells that regulates developmental and pathological angiogenesis.
- Mutations in endoglin lead to hereditary hemorrhagic telangiectasia—a disease characterized by arteriovenous malformations (AVMs).
- How endoglin regulates quiescent vasculature in adult life is not yet understood.

## References

[1] Castonguay R, Werner ED, Matthews RG, Presman E, Mulivor AW, Solban N, Sako D, Pearsall RS, Underwood KW, Seehra J (2011). Soluble endoglin specifically binds bone morphogenetic proteins 9 and 10 via its orphan domain, inhibits blood vessel formation, and suppresses tumor growth.. J Biol Chem.

[2] Saito T, Bokhove M, Croci R, Zamora-Caballero S, Han L, Letarte M, de Sanctis D, Jovine L (2017). Structural basis of the human endoglin-BMP9 interaction: insights into BMP signaling and HHT1.. Cell Rep.

[3] Lawera A, Tong Z, Thorikay M, Redgrave RE, Cai J, van Dinther M, Morrell NW, Afink GB, Charnock-Jones DS, Arthur HM (2019). Role of soluble endoglin in BMP9 signaling.. Proc Natl Acad Sci U S A.

[4] Torsney E, Charlton R, Parums D, Collis M, Arthur HM (2002). Inducible expression of human endoglin during inflammation and wound healing in vivo.. Inflamm Res.

[5] Lastres P, Bellon T, Cabañas C, Sanchez-Madrid F, Acevedo A, Gougos A, Letarte M, Bernabeu C (1992). Regulated expression on human macrophages of endoglin, an arg-gly-asp-containing surface antigen.. Eur J Immunol.

[6] Redgrave RE, Tual-Chalot S, Davison BJ, Singh E, Hall D, Amirrasouli MM, Gilchrist D, Medvinsky A, Arthur HM (2017). Cardiosphere-derived cells require endoglin for paracrine-mediated angiogenesis.. Stem Cell Reports.

[7] Lebrin F, Goumans MJ, Jonker L, Carvalho RL, Valdimarsdottir G, Thorikay M, Mummery C, Arthur HM, ten Dijke P (2004). Endoglin promotes endothelial cell proliferation and TGF-beta/ALK1 signal transduction.. EMBO J.

[8] Jin Y, Muhl L, Burmakin M, Wang Y, Duchez AC, Betsholtz C, Arthur HM, Jakobsson L (2017). Endoglin prevents vascular malformation by regulating flow-induced cell migration and specification through VEGFR2 signalling.. Nat Cell Biol.

[9] Sugden WW, Meissner R, Aegerter-Wilmsen T, Tsaryk R, Leonard EV, Bussmann J, Hamm MJ, Herzog W, Jin Y, Jakobsson L (2017). Endoglin controls blood vessel diameter through endothelial cell shape changes in response to haemodynamic cues.. Nat Cell Biol.

[10] Guttmacher AE, Marchuk DA, White RI (1995). Hereditary hemorrhagic telangiectasia.. N Engl J Med.

[11] Mahmoud M, Allinson KR, Zhai Z, Oakenfull R, Ghandi P, Adams RH, Fruttiger M, Arthur HM (2010). Pathogenesis of arteriovenous malformations in the absence of endoglin.. Circ Res.

[12] Garrido-Martin EM, Nguyen HL, Cunningham TA, Choe SW, Jiang Z, Arthur HM, Lee YJ, Oh SP (2014). Common and distinctive pathogenetic features of arteriovenous malformations in hereditary hemorrhagic telangiectasia 1 and hereditary hemorrhagic telangiectasia 2 animal models–brief report.. Arterioscler Thromb Vasc Biol.

[13] Tual-Chalot S, Oh SP, Arthur HM (2015). Mouse models of hereditary hemorrhagic telangiectasia: recent advances and future challenges.. Front Genet.

[14] Choi EJ, Chen W, Jun K, Arthur HM, Young WL, Su H (2014). Novel brain arteriovenous malformation mouse models for type 1 hereditary hemorrhagic telangiectasia.. PLoS One.

[15] Arthur HM, Ure J, Smith AJ, Renforth G, Wilson DI, Torsney E, Charlton R, Parums DV, Jowett T, Marchuk DA (2000). Endoglin, an ancillary TGFbeta receptor, is required for extraembryonic angiogenesis and plays a key role in heart development.. Dev Biol.

[16] Venkatesha S, Toporsian M, Lam C, Hanai J, Mammoto T, Kim YM, Bdolah Y, Lim KH, Yuan HT, Libermann TA (2006). Soluble endoglin contributes to the pathogenesis of preeclampsia.. Nat Med.

[17] Blázquez-Medela AM, García-Ortiz L, Gómez-Marcos MA, Recio-Rodríguez JI, Sánchez-Rodríguez A, López-Novoa JM, Martínez-Salgado C (2010). Increased plasma soluble endoglin levels as an indicator of cardiovascular alterations in hypertensive and diabetic patients.. BMC Med.

[18] Blaha M, Cermanova M, Blaha V, Jarolim P, Andrys C, Blazek M, Maly J, Smolej L, Zajic J, Masin V (2008). Elevated serum soluble endoglin (sCD105) decreased during extracorporeal elimination therapy for familial hypercholesterolemia.. Atherosclerosis.

[19] Kapur NK, Heffernan KS, Yunis AA, Parpos P, Kiernan MS, Sahasrabudhe NA, Kimmelstiel CD, Kass DA, Karas RH, Mendelsohn ME (2010). Usefulness of soluble endoglin as a noninvasive measure of left ventricular filling pressure in heart failure.. Am J Cardiol.

[20] Redgrave RE, Tual-Chalot S, Davison BJ, Greally E, Santibanez-Koref M, Schneider JE, Blamire AM, Arthur HM (2016). Using MRI to predict future adverse cardiac remodelling in a male mouse model of myocardial infarction.. Int J Cardiol Heart Vasc.

[21] Tual-Chalot S, Mahmoud M, Allinson KR, Redgrave RE, Zhai Z, Oh SP, Fruttiger M, Arthur HM (2014). Endothelial depletion of Acvrl1 in mice leads to arteriovenous malformations associated with reduced endoglin expression.. PLoS One.

[22] Mahmoud M, Borthwick GM, Hislop AA, Arthur HM (2009). Endoglin and activin receptor-like-kinase 1 are co-expressed in the distal vessels of the lung: implications for two familial vascular dysplasias, HHT and PAH.. Lab Invest.

[23] Shovlin CL (2010). Hereditary haemorrhagic telangiectasia: pathophysiology, diagnosis and treatment.. Blood Rev.

[24] Buscarini E, Danesino C, Olivieri C, Lupinacci G, Zambelli A (2005). Liver involvement in hereditary haemorrhagic telangiectasia or rendu-osler-weber disease.. Dig Liver Dis.

[25] Kline TL, Knudsen BE, Anderson JL, Vercnocke AJ, Jorgensen SM, Ritman EL (2014). Anatomy of hepatic arteriolo-portal venular shunts evaluated by 3D micro-CT imaging.. J Anat.

[26] Sadick H, Riedel F, Naim R, Goessler U, Hörmann K, Hafner M, Lux A (2005). Patients with hereditary hemorrhagic telangiectasia have increased plasma levels of vascular endothelial growth factor and transforming growth factor-beta1 as well as high ALK1 tissue expression.. Haematologica.

[27] Murphy CL, Thoms BL, Vaghjiani RJ, Lafont JE (2009). Hypoxia. HIF-mediated articular chondrocyte function: prospects for cartilage repair.. Arthritis Res Ther.

[28] da Rocha RC, Chopard RP (2004). Nutrition pathways to the symphysis pubis.. J Anat.

[29] David L, Mallet C, Keramidas M, Lamandé N, Gasc JM, Dupuis-Girod S, Plauchu H, Feige JJ, Bailly S (2008). Bone morphogenetic protein-9 is a circulating vascular quiescence factor.. Circ Res.

[30] Scharpfenecker M, van Dinther M, Liu Z, van Bezooijen RL, Zhao Q, Pukac L, Löwik CW, ten Dijke P (2007). BMP-9 signals via ALK1 and inhibits bFGF-induced endothelial cell proliferation and VEGF-stimulated angiogenesis.. J Cell Sci.

[31] Wang X, Abraham S, McKenzie JAG, Jeffs N, Swire M, Tripathi VB, Luhmann UFO, Lange CAK, Zhai Z, Arthur HM (2013). LRG1 promotes angiogenesis by modulating endothelial TGF-β signalling.. Nature.

[32] Anderberg C, Cunha SI, Zhai Z, Cortez E, Pardali E, Johnson JR, Franco M, Páez-Ribes M, Cordiner R, Fuxe J (2013). Deficiency for endoglin in tumor vasculature weakens the endothelial barrier to metastatic dissemination.. J Exp Med.

[33] Tian H, Huang JJ, Golzio C, Gao X, Hector-Greene M, Katsanis N, Blobe GC (2018). Endoglin interacts with VEGFR2 to promote angiogenesis.. FASEB J.

[34] Reddy YNV, Obokata M, Dean PG, Melenovsky V, Nath KA, Borlaug BA (2017). Long-term cardiovascular changes following creation of arteriovenous fistula in patients with end stage renal disease.. Eur Heart J.

[35] Nassiri N, Thomas J, Rahimi S (2015). Fibrodysplastic implications for transvenous embolization of a high-flow pelvic arteriovenous malformation in osler-weber-rendu syndrome.. J Vasc Surg Cases.

[36] Mookadam M, Jiamsripong P, Mookadam F (2010). High-output heart failure secondary to large pelvic arteriovenous malformation.. Am J Med Sci.

[37] Calligaro KD, Sedlacek TV, Savarese RP, Carneval P, DeLaurentis DA (1992). Congenital pelvic arteriovenous malformations: long-term follow-up in two cases and a review of the literature.. J Vasc Surg.

[38] Okada M, Kato M, Uchida K, Sufu Y, Okuda S, Yano M, Matsunaga N (2015). Transcatheter and percutaneous procedures for huge pelvic arteriovenous malformations causing high-output heart failure.. J Cardiol Cases.

[39] Snellings DA, Gallione CJ, Clark DS, Vozoris NT, Faughnan ME, Marchuk DA (2019). Somatic mutations in vascular malformations of hereditary hemorrhagic telangiectasia result in bi-allelic loss of ENG or ACVRL1.. Am J Hum Genet.

[40] Pfander D, Körtje D, Zimmermann R, Weseloh G, Kirsch T, Gesslein M, Cramer T, Swoboda B (2001). Vascular endothelial growth factor in articular cartilage of healthy and osteoarthritic human knee joints.. Ann Rheum Dis.

[41] Zelzer E, Olsen BR (2005). Multiple roles of vascular endothelial growth factor (VEGF) in skeletal development, growth, and repair.. Curr Top Dev Biol.

[42] Flieger D, Hainke S, Fischbach W (2006). Dramatic improvement in hereditary hemorrhagic telangiectasia after treatment with the vascular endothelial growth factor (VEGF) antagonist bevacizumab.. Ann Hematol.

[43] Mitchell A, Adams LA, MacQuillan G, Tibballs J, vanden Driesen R, Delriviere L (2008). Bevacizumab reverses need for liver transplantation in hereditary hemorrhagic telangiectasia.. Liver Transpl.

[44] Dupuis-Girod S, Ginon I, Saurin JC, Marion D, Guillot E, Decullier E, Roux A, Carette MF, Gilbert-Dussardier B, Hatron PY (2012). Bevacizumab in patients with hereditary hemorrhagic telangiectasia and severe hepatic vascular malformations and high cardiac output.. JAMA.

[45] Thalgott JH, Dos-Santos-Luis D, Hosman AE, Martin S, Lamandé N, Bracquart D, Srun S, Galaris G, de Boer HC, Tual-Chalot S (2018). Decreased expression of vascular endothelial growth factor receptor 1 contributes to the pathogenesis of hereditary hemorrhagic telangiectasia type 2.. Circulation.

[46] Crist AM, Lee AR, Patel NR, Westhoff DE, Meadows SM (2018). Vascular deficiency of Smad4 causes arteriovenous malformations: a mouse model of hereditary hemorrhagic telangiectasia.. Angiogenesis.

[47] Nikolaev SI, Vetiska S, Bonilla X, Boudreau E, Jauhiainen S, Rezai Jahromi B, Khyzha N, DiStefano PV, Suutarinen S, Kiehl TR (2018). Somatic activating KRAS mutations in arteriovenous malformations of the brain.. N Engl J Med.

[48] Baeyens N, Larrivée B, Ola R, Hayward-Piatkowskyi B, Dubrac A, Huang B, Ross TD, Coon BG, Min E, Tsarfati M (2016). Defective fluid shear stress mechanotransduction mediates hereditary hemorrhagic telangiectasia.. J Cell Biol.

[49] Bidart M, Ricard N, Levet S, Samson M, Mallet C, David L, Subileau M, Tillet E, Feige JJ, Bailly S (2012). BMP9 is produced by hepatocytes and circulates mainly in an active mature form complexed to its prodomain.. Cell Mol Life Sci.

[50] Tillet E, Ouarné M, Desroches-Castan A, Mallet C, Subileau M, Didier R, Lioutsko A, Belthier G, Feige JJ, Bailly S (2018). A heterodimer formed by bone morphogenetic protein 9 (BMP9) and BMP10 provides most BMP biological activity in plasma.. J Biol Chem.

[51] Alsina-Sanchís E, García-Ibáñez Y, Figueiredo AM, Riera-Domingo C, Figueras A, Matias-Guiu X, Casanovas O, Botella LM, Pujana MA, Riera-Mestre A (2018). ALK1 loss results in vascular hyperplasia in mice and humans through PI3K activation.. Arterioscler Thromb Vasc Biol.

[52] Ola R, Dubrac A, Han J, Zhang F, Fang JS, Larrivée B, Lee M, Urarte AA, Kraehling JR, Genet G (2016). PI3 kinase inhibition improves vascular malformations in mouse models of hereditary haemorrhagic telangiectasia.. Nat Commun.

[53] Park SO, Wankhede M, Lee YJ, Choi EJ, Fliess N, Choe SW, Oh SH, Walter G, Raizada MK, Sorg BS (2009). Real-time imaging of de novo arteriovenous malformation in a mouse model of hereditary hemorrhagic telangiectasia.. J Clin Invest.

[54] Morine KJ, Qiao X, Paruchuri V, Aronovitz MJ, Mackey EE, Buiten L, Levine J, Ughreja K, Nepali P, Blanton RM (2017). Conditional knockout of activin like kinase-1 (ALK-1) leads to heart failure without maladaptive remodeling.. Heart Vessels.

[55] Duffy AG, Ma C, Ulahannan SV, Rahma OE, Makarova-Rusher O, Cao L, Yu Y, Kleiner DE, Trepel J, Lee MJ (2017). Phase I and Preliminary Phase II Study of TRC105 in Combination with Sorafenib in Hepatocellular Carcinoma.. Clin Cancer Res.

[56] Rosen LS, Hurwitz HI, Wong MK, Goldman J, Mendelson DS, Figg WD, Spencer S, Adams BJ, Alvarez D, Seon BK (2012). A phase I first-in-human study of TRC105 (anti-endoglin antibody) in patients with advanced cancer.. Clin Cancer Res.

[57] Gomes AC, Falcão-Pires I, Pires AL, Brás-Silva C, Leite-Moreira AF (2013). Rodent models of heart failure: an updated review.. Heart Fail Rev.

[58] Reddy YNV, Melenovsky V, Redfield MM, Nishimura RA, Borlaug BA (2016). High-output heart failure: a 15-year experience.. J Am Coll Cardiol.

