# Supplementary material for: Loss of Endothelial Endoglin Promotes High-Output Heart Failure Through Peripheral Arteriovenous Shunting Driven by VEGF Signaling
Source: Circ Res. 2019 Dec 6;126(2):243–57. doi: 10.1161/CIRCRESAHA.119.315974 (PMC6970547; doi:10.1161/CIRCRESAHA.119.315974)
Supplement: Supplementary file 1 [file res-126-243-s001.pdf]

**Supplementary Material**

**Materials and Methods**

ELISA

Circulating VEGF was measured in 10 µl mouse serum by Quantikine ELISA (MMV00; R&D) following manufacturer's instructions.

Oxygen Saturation and blood parameters

Adult mice were anesthetised using isoflurane mixed with medical air, and oxygen saturation of a hind limb was assessed by pulse oxymetry (MouseOx; Starr Life Sciences Corp.). Blood chemistry parameters were obtained using blood harvested by left ventricular cardiac puncture from terminally anesthetised adult mice (one month after endoglin knockdown) and analysed on a CC8+ cartridge (Abaxis) using an i-STAT portable reader (Abbott Laboratories, Princeton, NJ).

Telemetry and Cardiac Conductance

A small pressure catheter connected to a radio telemetry device (model TA11PA-C10; DSI) was inserted in the aorta via the carotid artery in anaesthetised mice. Continuous blood pressure measurements were made following full recovery from surgery (at least 7 days) and data analysed using Ponemah software. To measure cardiac ventricular pressure, a small catheter (SPR-1045, Millar) was placed in either the left or right ventricle in terminally anaesthetised mice and data recorded using an MPVS-Ultra Single Segment Foundation System (AD instruments) and analysed using labchart software (version 8).

Microvessel perfusion

To investigate right to left shunting, 2 $x10^5$ fluorescent 15$\mu m^3$ microspheres (Invitrogen) suspended in 100 µl PBS were injected into the tail vein of anaesthetised mice (isoflurane), whilst to investigate left to right shunting 5 $x10^4$ fluorescent 45 $\mu m^3$ microspheres (Polysciences, Inc.) were injected into left cardiac ventricle. Mice were killed by injection of an overdose of pentobarbital for 1 min and fluorescent beads trapped in the tissue vasculature were examined using a Zeiss Axioimager microscope.

A separate group of animals were used to visualise the vasculature following perfusion with Microfil (Flow Tech Inc.) or latex (Connecticut valley Biological Supply Co), under terminal anaesthesia (isoflurane). Mice were initially injected i.p. with heparin (200 µl of 2000U/ml)

at 30 minutes prior to systemic perfusion with 10 ml 2000 U/ml heparin at 1.25 ml/min to remove blood, subsequently vascular tracer was injected into the aorta at 0.4 ml/min. Tissues were left in situ for 1hr at room temperature and organs were harvested and fixed in 10% formalin. For whole-mount imaging, tissue was dehydrated in methanol and cleared with organic solvent (benzyl alcohol/benzyl benzoate, 1:1; Sigma-Aldrich).

Micro-CT

Microfil perfused tissue samples were placed inside a 10 ml syringe and sealed to prevent dehydration. The Syringe was placed into a polystyrene tube and then positioned onto the CT bed for imaging. All samples were acquired using Skyscan 1176, Bruker-microCT at an isotropic resolution of 9 μm using the following parameters: 50kV, 500 μA, 1010 ms exposure time, 0.5mm Aluminium filter, 0.3° rotation step, 4 frame averaging. Total scan duration was 1 hour and 17 minutes. For image analysis raw projection data were reconstructed using GPUReconServer (Bruker-microCT) and NRecon Reconstruction 64-bit software (Bruker-microCT). Data was analysed using CT Analyser (Version 1.17.7 Bruker microCT). Adaptive thresholding and 3D analysis function was used to determine structural thickness. CTvox (Version 3.3.0 Bruker microCT) was used for 3D volume rendering and data visualisation .All images were converted to 8 bit grayscale and segmented in microscopy image browser (MIB) using threshold selection. The default smoothing settings were used to smooth the segmented vessels (2D, Kernel=5, Z=5, ∑=5) and Amira Software (Thermo scientific) was used to render the surface.

qPCR

RNA was prepared from heart tissue as previously described [1] using a Qiagen RNeasyFibrous Tissue Mini Kit (Qiagen). Tetro cDNA synthesis kit(Bioline) was used to prepare cDNA and gene expression was determined using Taq polymerase and Taqman probes (Thermofisher Scientific) for Acta1 (Mm00808218), Anp (Mm01255747), Bnp (Mm01255770), Eng (Mm00468252), TGFb1 (Mm01178820), Vegfa (Mm00437306), Gapdh(Mm99999915) and Hprt (Mm00446968) on a Quantstudio 7 Flex Real-Time PCR system. Data were analyzed using the comparative Ct method to determine relative gene expression.

Western Blot

Mouse lung endothelial cells (MLECs) were prepared as previously described[2]. Briefly, lung ECs were purified using anti-CD31 antibody (553370, BD Biosciences) conjugated to

Dynabeads (Invitrogen), and maintained in Endothelial Cell Basal Medium MV2 (PromoCell). Endoglin knockout cells were generated by temporary addition of 1 µM 4-hydroxytamoxifen (4-OHT) (Sigma) for 48hr, and cells cultured for a further 48-72h before being used in experiments. Cells were serum starved for 1h (0.5% serum) with all MV2 supplements removed and then stimulated with 10ng/ml VEGF (R&D) for 0, 5, 10, and 30min. Proteins in SDS sample were separated on 10% acrylamide gel and transferred onto polyvinylidene fluoride membrane before blocking with 5% powdered milk/Tris-buffered saline/Tween 20 and incubating with primary antibody to detect Endoglin (AF1097, R&D); phospho-VEGFR2$^{Tyr1175}$(2478, Cell Signaling), VEGFR2 (9698, Cell Signaling), phospho-AKT$^{Ser473}$ (4060, Cell Signaling), AKT (4691, Cell Signaling), phospho- ERK1/2$^{Thr202/Tyr204}$ (4377, Cell Signaling), ERK1/2 (4695, Cell Signaling), and α-tubulin (T9026, Sigma). Detection was achieved using dye-conjugated secondary antibodies IRDye 800CW Goat Anti-Rabbit (926-32211, LiCor) and IRDye 680RD Goat Anti-Mouse (926-68070, LiCor) followed by scanning on an Odyssey CLx imager and analysis using Odyssey software.

Histology and immunofluorescent staining

For cardiomyocyte size analysis, 10 µm transverse heart sections were incubated with 20µg/ml fluorescein conjugated wheat germ agglutinin (Vector Laboratories, #FL-1021) in PBS for 1 hour at room temperature, before washing in PBS and imaging using a Zeiss Axioimager and Axiocam digital camera. Average cardiomyocyte area was measured from at least 30 cardiomyocytes per heart using ImageJ (NIH). Mouse knee joints were cleaned from skin & muscle, fixed in 4% formalin for 24h and decalcificied in Formical-2000 for 16 hours at room temperature, prior to processing to paraffin. Microtome sections (10 µm) were stained with Masson's trichrome using the Trichrome Stain Kit (Sigma), according to manufacturer's instructions. Immunofluorescent (IF) staining of organ and pubic symphysis cryosections (10 µm) was performed as previously described[3, 4] using primary antibodies to Alk1 (AF770, R&D), ASMA (A2547, Sigma), CD31 (553370, BD Biosciences), Endoglin (AF1097, R&D) and pERK (4377, Cell Signaling) with detection using secondary antibodies conjugated with Alexa fluors. A secondary antibody only control was used in each case to ensure specificity of immunostaining. Masson's trichrome staining used the Trichrome Stain Kit (Sigma), according to manufacturer's instructions.

For whole mount tissue staining, mice were anaesthetised and perfused with 20 ml of HEPES followed by cervical dislocation. The pelvic region was dissected out and fixed in 4% PFA at

4°C for 10 hours. Following removal of muscles ventral to the pubic symphysis cartilage, tissue samples were incubated in phosphate buffered saline (PBS) with 0.5% triton X-100 and 1.5% BSA for 3 hours at room temperature and then with primary antibodies: mouse anti α-smooth muscle actin (C6198, Sigma, 1:400); goat anti-mouse CD31 (AF3628, R&D Systems, 1:400); chicken anti-GFP (ab13970, Abcam, 1:1000); rabbit anti-ERG (ab92513, Abcam, 1:200) diluted in the same solution overnight at 4°C with agitation. After 3 washes at room temperature, samples were incubated with secondary antibodies conjugated with Alexa Fluor dyes donkey anti-rabbit AF 555 (Invitrogen, A31572), donkey anti-rabbit AF 647 (Life technologies, A31573), donkey anti-chicken DL 488 (Jackson, 703-545-155) or donkey anti-goat Cy3 (Jackson, 705-166-147) overnight at 4°C, washed 3 times and mounted to slides for imaging. Samples were imaged using a stereomicroscope (Zeiss Lumar.V12) or confocal microscopes (LSM 700, Carl Zeiss AG or Leica SP8, Leica Microsystems).

Magnetic Resonance Imaging

Cardiac function was analysed by MRI using a horizontal bore 7 Tesla microimaging system (Varian Inc, Paulo Alto, CA, USA) equipped with a 12-cm microimaging gradient insert (40 gauss/cm) as previously described[1, 5]. Mice were anaesthetised with isoflurane and positioned on a custom built sled with integrated electrocardiographic, respiratory and cutaneous temperature probes (Dazai Research Instruments, Toronto, Canada) connected to MR compatible monitoring equipment (SA Instruments Inc. Edison, NJ, USA). The sled was placed in a 39mm quadrature birdcage volume coil (Rapid Biomedical, GmbH) and global cardiac function was measured using an ECG triggered, respiratory gated gradient echo (FLASH) cine MR sequence. Images were analysed using Image J software to determine cardiac parameters. Heart wall dimensions were measured at 3 sites along both the right and left free ventricular wall.

Data Presentation and Statistical Analysis

All data were analysed using Graphpad Prism software and are presented as mean ± standard error for n biological replicates. Normality was tested using the Shapiro-Wilk test, where n numbers were sufficient, and used to inform the choice of parametric versus non-parametric statistical tests as follows. Two experimental groups were compared using Students unpaired t test or Mann Whitney test, whilst multiple groups were tested using ANOVA (one-way or two-way) or Kruskal-Wallis test. Bonferroni corrections for multiple comparisons were

applied for all data analysed by ANOVA. Differences were considered significant where $p<0.05$. An across experiment multiple test correction was not performed due to the different endpoints of each experiment. Representative figures were selected on the basis of their proximity to the mean/median of the full range of data points.

## Online Figure I

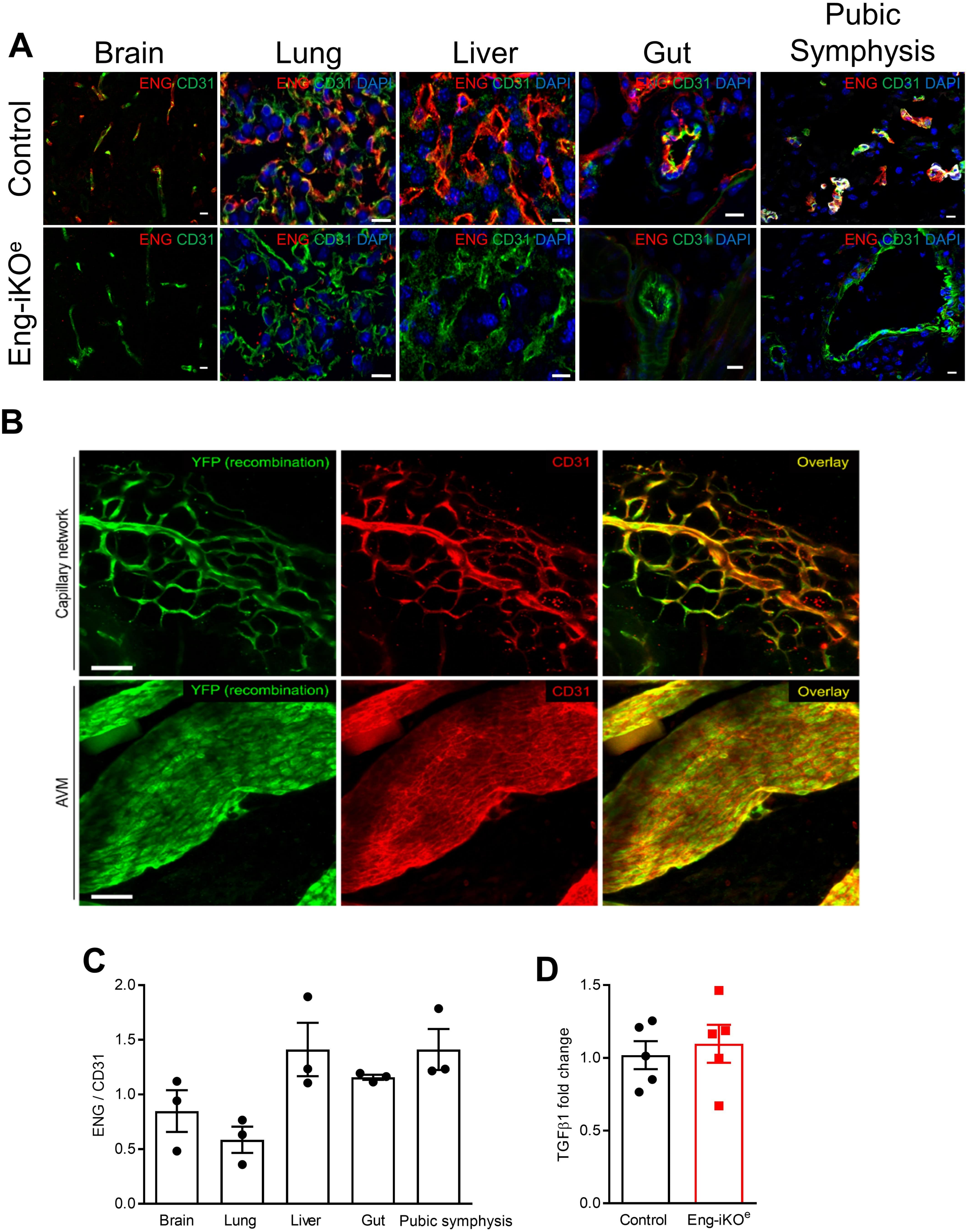

**Online Figure I. Endoglin knockdown in Eng-iKO$^e$ mice.**

**A**, Immunostaining with anti-ENG (=anti-CD105) antibody reveals ENG protein is expressed in endothelial cells of brain, lung, liver, gut and pubic symphysis in controls, and is efficiently reduced in Eng-iKO$^e$ mice. Co-staining with anti-CD31 is used to identify endothelial cells (scale bar=10µm).

**B,** In mice where *Gt(ROSA)26Sor*$^{tm3(CAG\text{-}EYFP)Hze}$ is used as a reporter for Cre recombination following tamoxifen treatment, YFP is expressed in almost 100% of CD31+ endothelial cells whether in capillary network or within the AVM, showing a high efficiency of Cre recombination.

**C,** ENG expression normalised to expression of the pan-endothelial marker CD31 is similar in the pubic symphysis compared with other tissues affected by HHT. Data were analysed using Kruskal-Wallis one way ANOVA (n=3).

**D,** Expression of TGFbeta1 does not change in the pubic symphysis after Eng knockdown. Data were analysed using Student’s t-test (n=5).

## Online Figure II

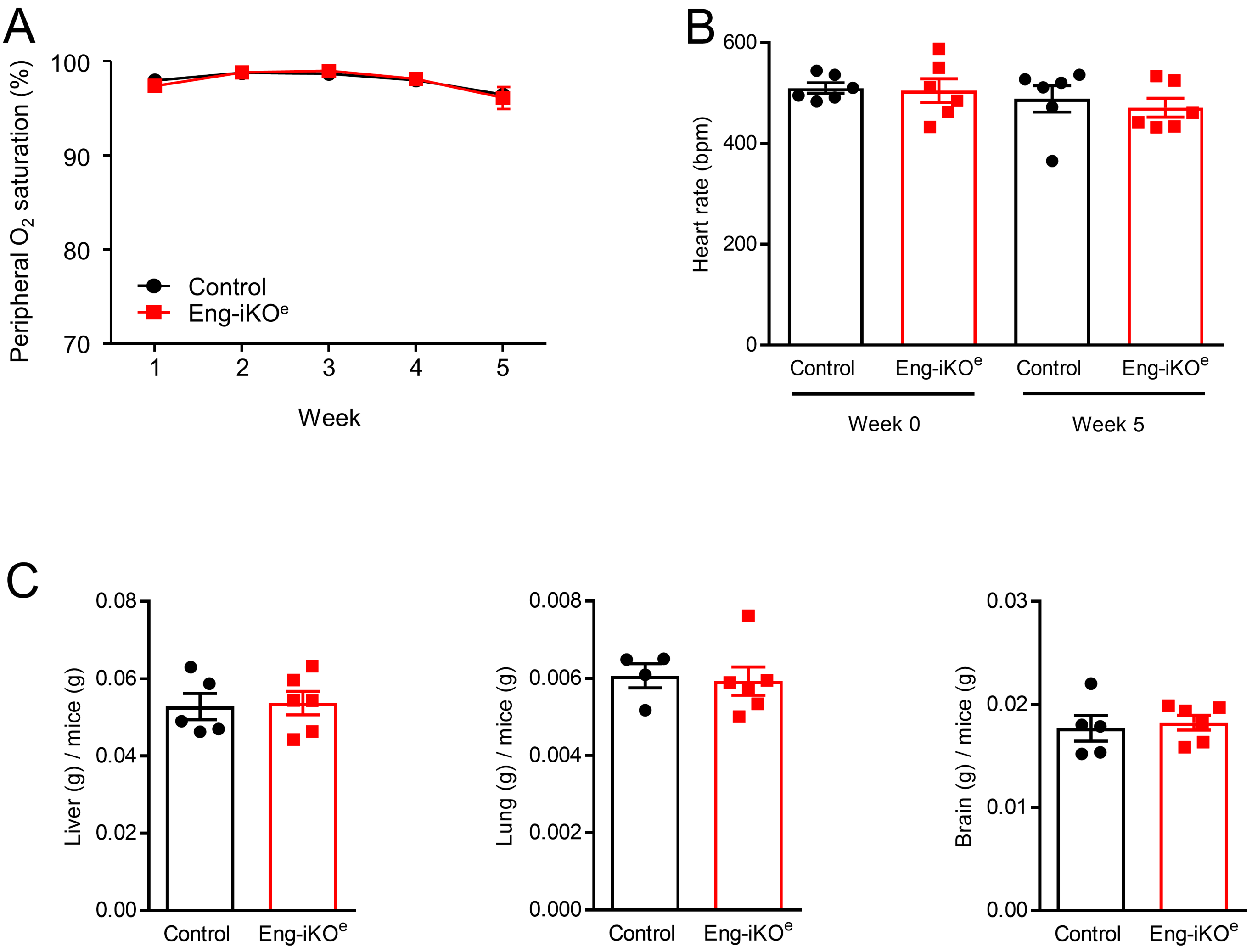


**Online Figure II. No change in peripheral oxygen saturation, heart rate or tissue oedema in Eng-iKO$^e$ Mice. A**, Oximetry was used to measure peripheral oxygen saturation on a weekly basis for 5 weeks after endoglin depletion. Mice were anaesthetised on medical air and the probe was placed across the mouse thigh No difference was observed betweenEng-iKOe and control mice; data was analysed using two way ANOVA with Bonferroni correction (n=3-4/group) . **B**, heart rate (under anaesthesia) was unchanged in Eng-iKO$^e$ mice compared with controls at 5 weeks after endoglin depletion. Data were analysed using two way ANOVA with Bonferroni correction (n=6/group). **C**, Weight of liver, lung and brain was similar in Eng-iKO$^e$ and control mice at 5 weeks after tamoxifen treatment consistent with a lack of tissue oedema Data were analysed using Student's t-test (n=4-6/group).

## Online Figure III

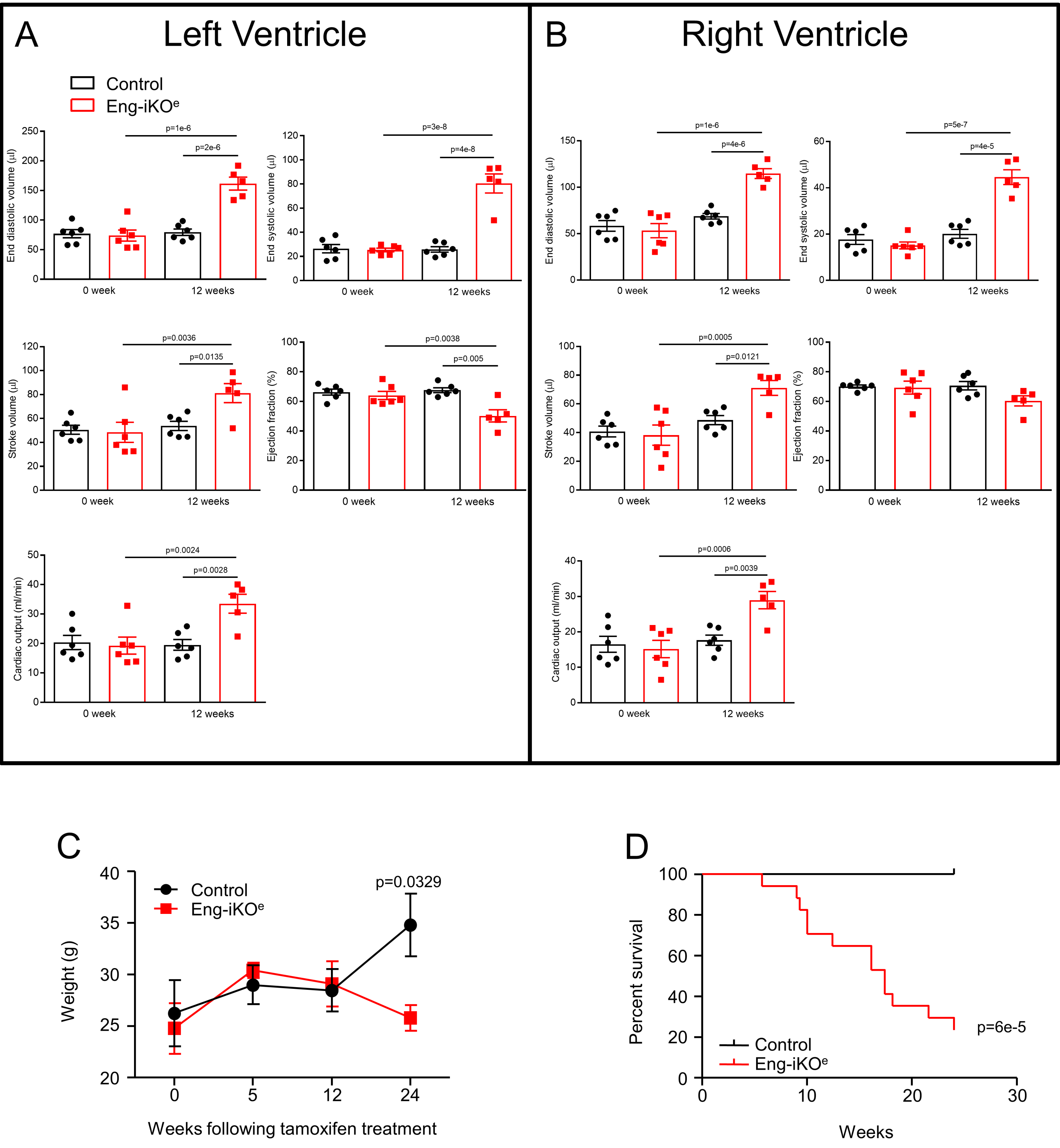

**Online Figure III. Major heart remodelling and mortality in Eng-iKO$^{e}$ mice. A** and **B**, MRI data shows a progressive increase in left and right ventricular end diastolic and end systolic volumes at 12 weeks after knockdown of endothelial endoglin. This was accompanied by an increase in cardiac output, and a significant decrease in ejection fraction compared with matched control group. Data were analysed using two way ANOVA with Bonferroni correction (n=5-6/group). **C**, There is no difference in the body weight of Eng-iKO$^{e}$ and control adult mice immediately following tamoxifen treatment. However Eng-iKO$^{e}$ mice show weight loss by 24 weeks. Data were analysed using two way ANOVA with Bonferroni correction (n=5-11/group). **D**, Kaplan Meier curve shows increased mortality in the absence of endothelial endoglin (n=16-17/group). Log-rank (Mantel-Cox) was used for comparison of survival curve.

## Online Figure IV

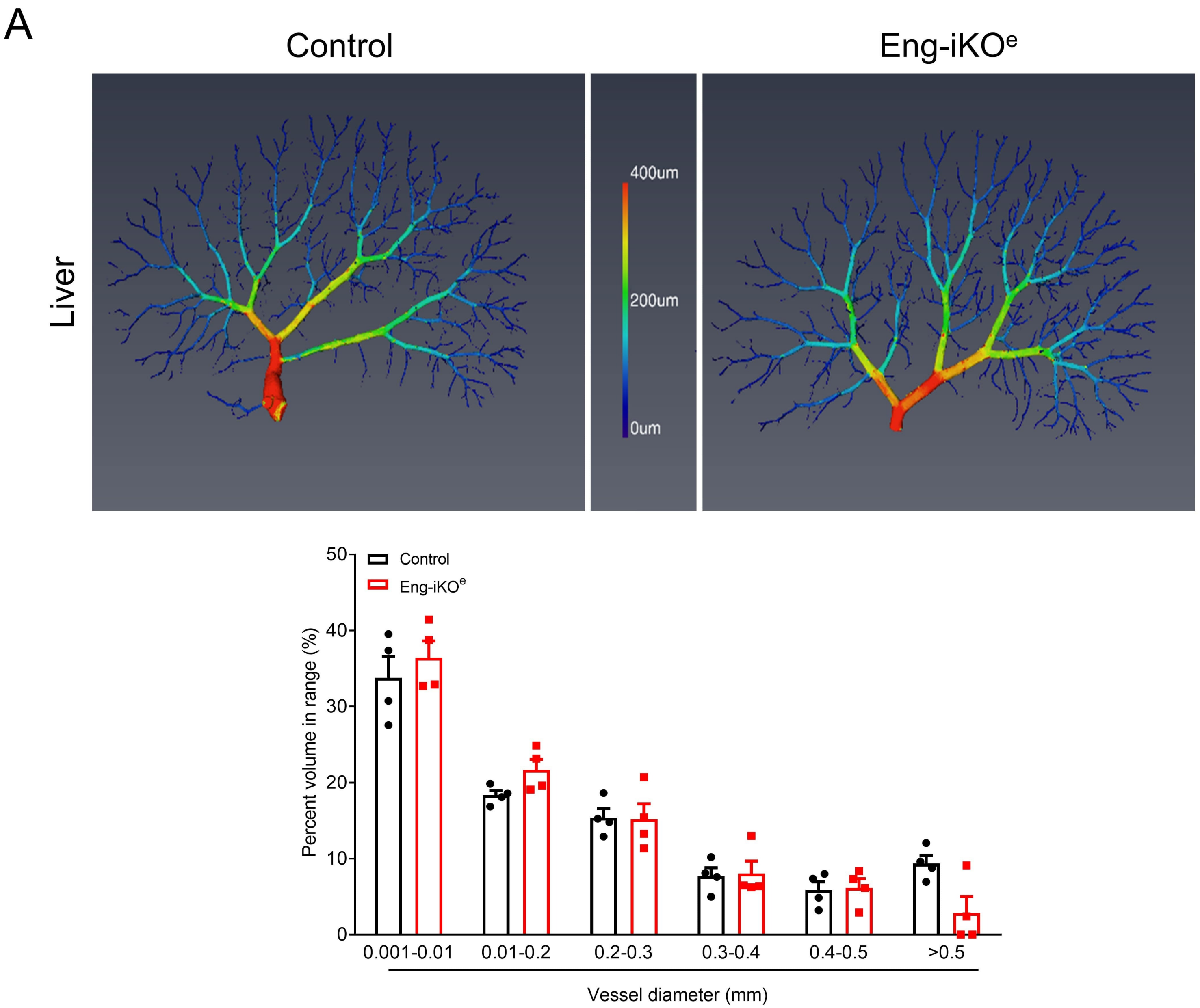


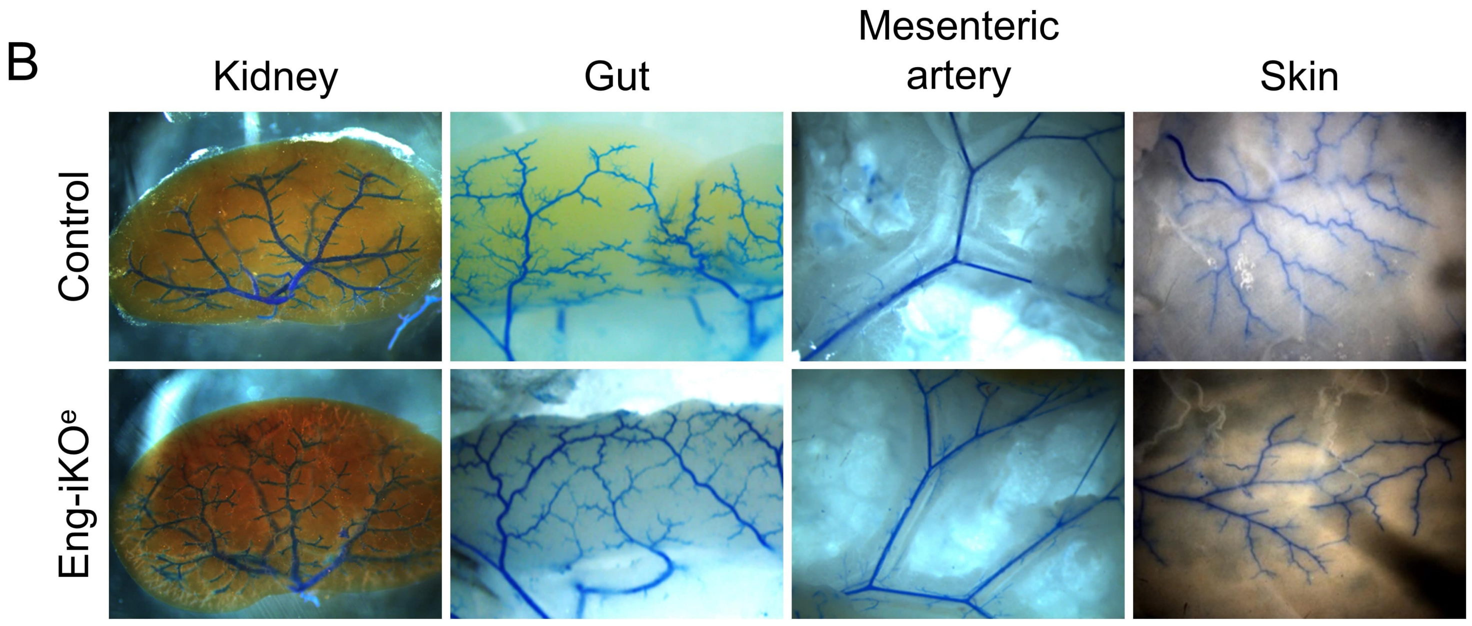

**Online Figure IV. Vascular perfusion of systemic organs. A**, The vasculature of the liver was analysed using microfil. There was no evidence of transfer of perfusate from arterial to venous vessels indicating an absence of AVMs. Microfil perfused livers were analysed using microCT and vessel size analysis showed similar distribution of vessel sizes in livers from control and Eng-iKO$^e$ mice (n=4/group). **B**, Vascular perfusion of systemic organs using latex illustrated the majority of the vessels lacked direct artery to venous connections in both control and Eng-iKO$^e$ mice. Data were analysed using two way ANOVA with Bonferoni correction (n=4/group).

## **Online Figure V**

A

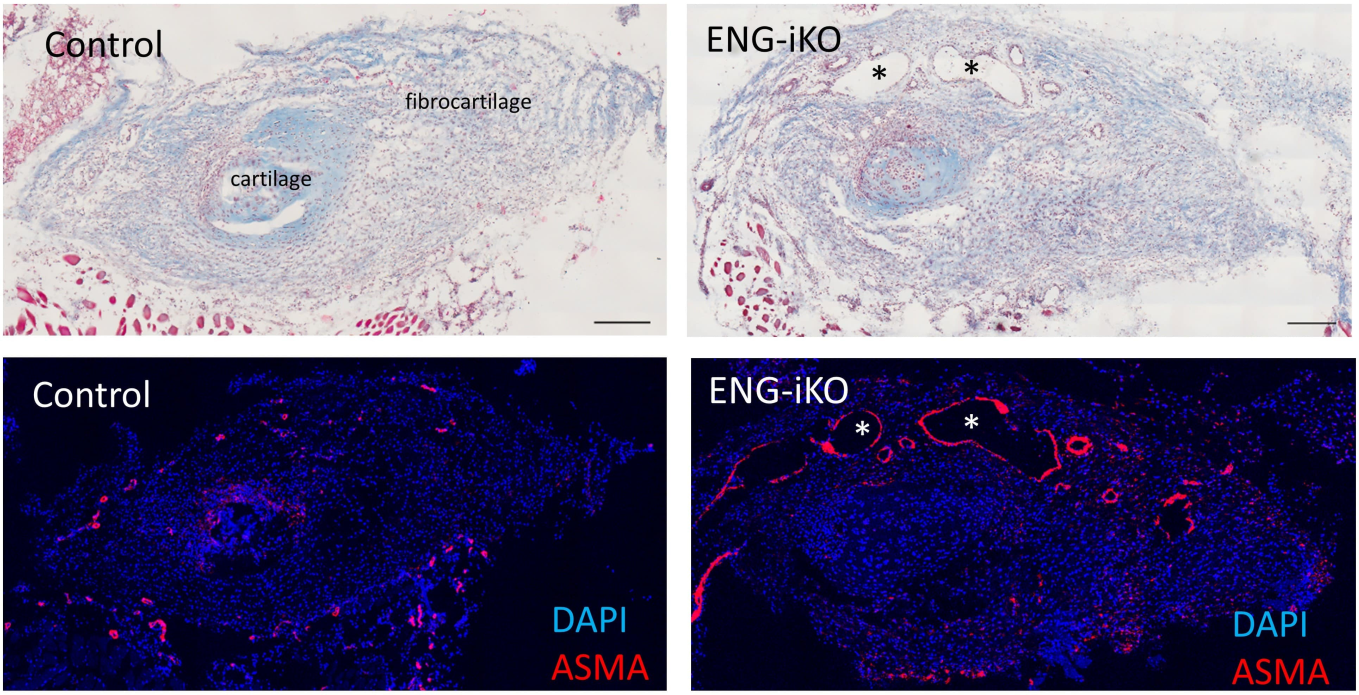


B

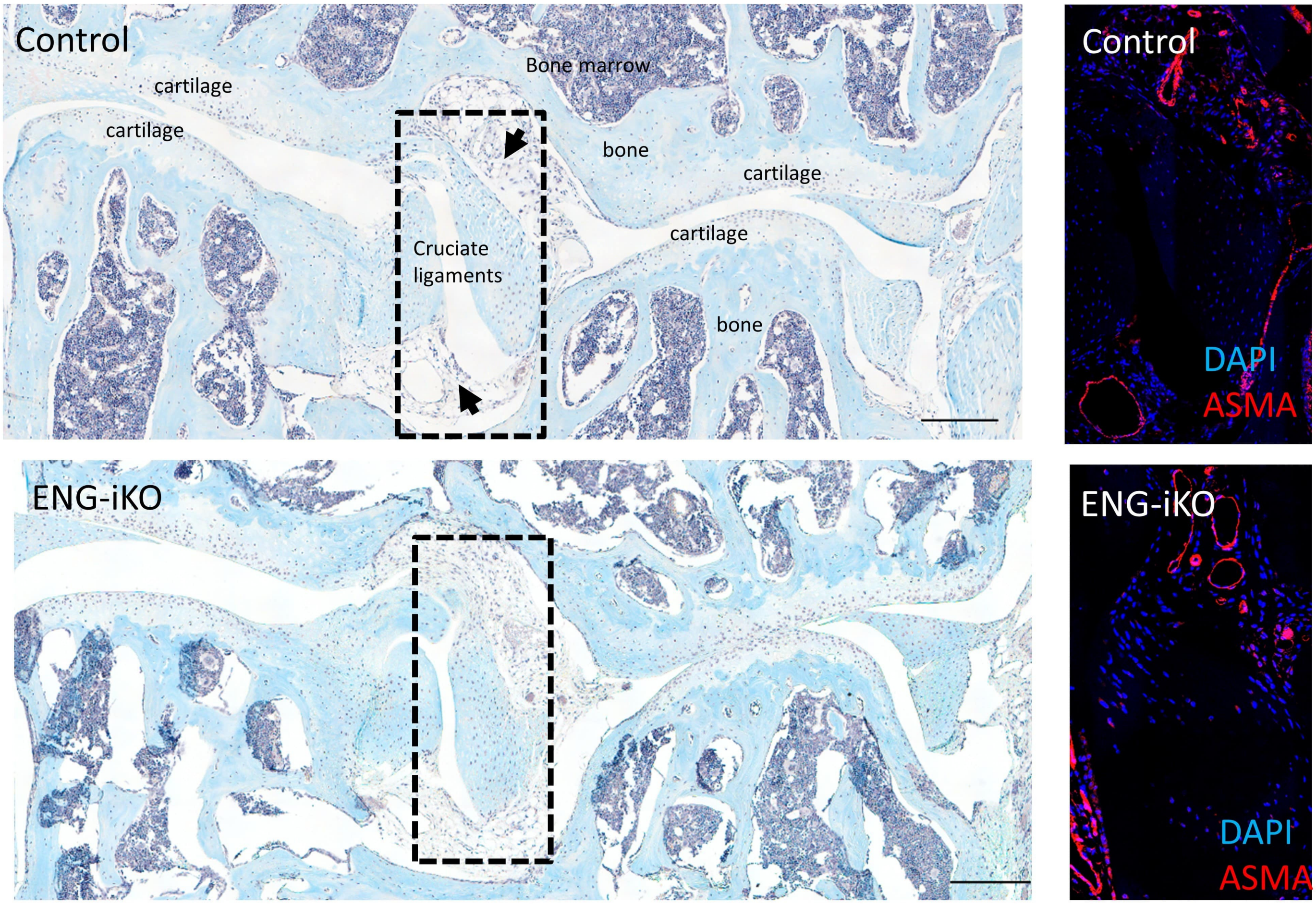

**Online Figure V. There is intimate association of blood vessels and cartilage within the pubic symphysis .**

**A**, Cartilage and fibrocartilage of the pubic symphysis appear blue following Masson's trichrome stain. Blood vessels visualised using asma staining (red) on a serial section are visible throughout the fibrocartilage in controls. Enlarged blood vessels (asterisks) can be see within the ventral fibrocartilage of the pubic symphysis in Eng-iKOe mice. Serial section shows the enlarged asma positive blood vessels in Eng-iKOe mice. Scale bar = 200µm

**B,** The knee joint of control and Eng-iKOe adult mice show similar organisation of articular cartilage, bone and bone marrow. Within the joint the most vascularised region surrounds the cruciate ligaments. This peri-ligament tissue (arrows) contrasts with the hypovascular nature of the ligaments themselves as well as the neighbouring articular cartilage. ASMA staining of vascular smooth muscle allows comparison of the knee vasculature and no differences are observed between control and Eng-iKOe mice. Scale bar = 200µm.

# Online Figure VI

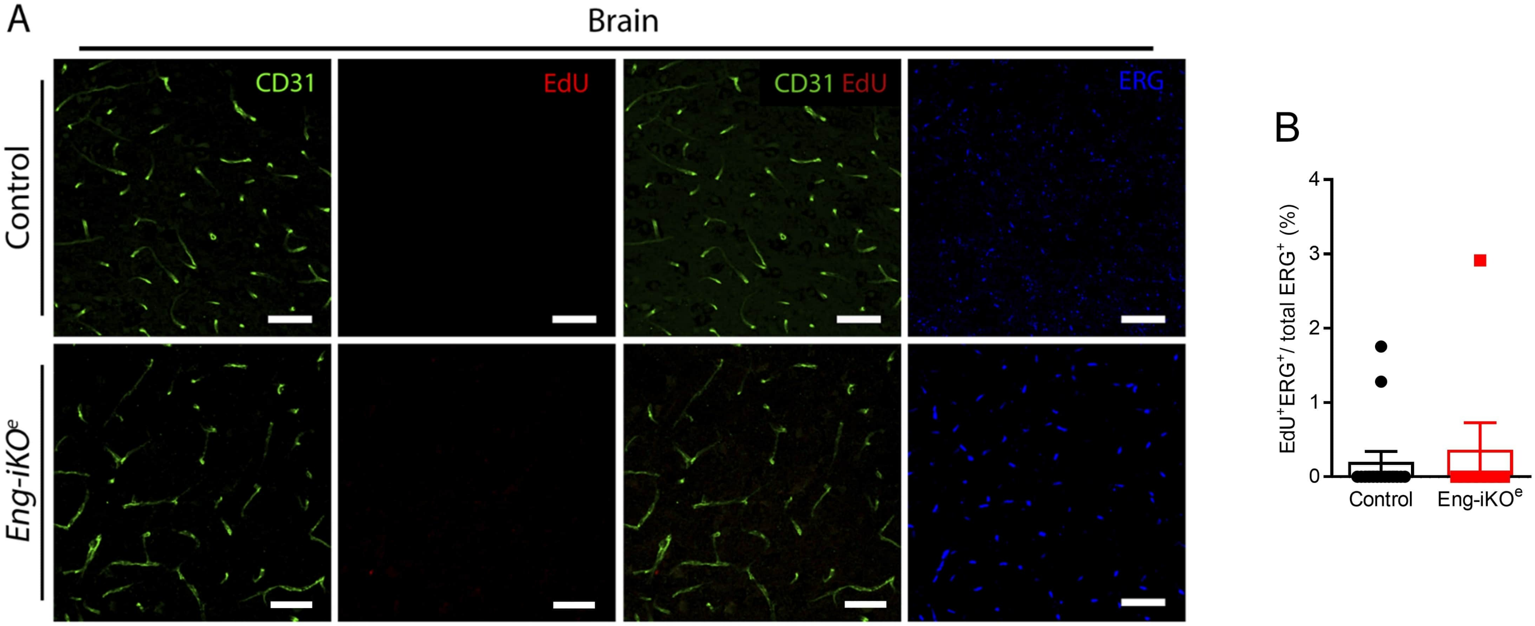


**Online Figure VI Endothelial cell proliferation is low in adult brain tissue**

A: Brain vessels were exposed to repeated EdU injections during two weeks and stained for CD31 (ECs, green), ERG (ECs, blue) and EdU (proliferating cells, red). Scale bars=50 µm. B:Quantification of EC proliferation in the brain showed that only 0.2 % and 0.4 % of ECs were proliferative in control (n =4) and Eng-iKOe (n=2) mice , respectively.

## Online Table I

**Loss of endothelial endoglin leads to increased left ventricular mass and increased cardiac output**

Longitudinal study of heart function in control and Eng-iKO$^{e}$ mice using cardiac MRI demonstrates a significant increase in left ventricular mass, stroke volume and cardiac output at 5 weeks following endothelial endoglin depletion. Data (shown as mean +/-SEM) were analysed by two way ANOVA with Bonferroni correction for multiple comparisons (n=6/group).

| Cardiac Parameters | Week 0 | Week 1 | Week 2 | Week 3 | Week 4 | Week 5 |
|---|---|---|---|---|---|---|
| **Mass (mg)** | | | | | | |
| Control | 115.8 ± 5 | 117.1 ± 3.2 | 121.1 ± 6.3 | 121.1 ± 4.1 | 128 ± 5.9 | 123.7 ± 2.8 |
| Eng-iKO$^{e}$ | 113.4 ± 6.8 | 121.1 ± 6.2 | 140.4 ± 9.3 | 145 ± 11 | 156.3 ± 12.3 | **168.1 ± 9.5 (p=0.0014)** |
| **Ejection fraction (%)** | | | | | | |
| Control | 79.2 ± 1.2 | 79.8 ± 0.9 | 77.6 ± 1.3 | 76.5 ± 0.7 | 76.4 ± 1.3 | 73.6 ± 2.1 |
| Eng-iKO$^{e}$ | 76 ± 1.6 | 77.9 ± 1.3 | 74.4 ± 1.6 | 75.4 ± 2.1 | 72.5 ± 1 | 69.9 ± 1 |
| **Heart rate (bpm)** | | | | | | |
| Control | 497.7 ± 5.8 | 481.5 ± 9.1 | 486.3 ± 8.7 | 483.5 ± 10.5 | 487 ± 8.7 | 484.8 ± 26.4 |
| Eng-iKO$^{e}$ | 501.8 ± 8.3 | 478.3 ± 7.4 | 490.5 ± 17.6 | 488.2 ± 20.8 | 490.2 ± 20.7 | 482.8 ± 17.7 |
| **Stroke volume (µl)** | | | | | | |
| Control | 56.2 ± 2.3 | 57 ± 1.5 | 57.4 ± 3.2 | 62.4 ± 1.9 | 64.1 ± 1.4 | 58.9 ± 0.7 |
| Eng-iKO$^{e}$ | 51.8 ± 3.4 | 57.1 ± 2.4 | 64.5 ± 2.6 | 73.8 ± 4.9 | 76 ± 7.6 | **80.2 ± 5.3 (p=0.006)** |
| **Cardiac output (ml/min)** | | | | | | |
| Control | 28 ± 1.2 | 27.4 ± 0.8 | 28 ± 1.8 | 30.2 ± 1.5 | 31.2 ± 0.7 | 28.6 ± 1.5 |
| Eng-iKO$^{e}$ | 26 ± 1.6 | 27.3 ± 0.9 | 31.6 ± 1.5 | 35.8 ± 2 | 36.7 ± 2.4 | **38.4 ± 2 (p=0.0088)** |

## Online Table II

**Cardiac pressures are significantly altered in both the left and right ventricles one week following loss of systemic endothelial endoglin.** Data analysed by Student's unpaired t-test.

| **Left Ventricle** | **Control (n=5)** | **Eng-iKO$^e$ (n=6)** |
|---|---|---|
| End-systolic pressure | 90.69 ± 3.5 | **77.83 ± 3.1 (p=0.023)** |
| End-diastolic pressure | 3.89 ± 0.31 | 4.6 ± 1.5 |
| dP/dT max | 8768 ± 1745 | 8302 ± 1226 |

| **Right Ventricle** | **Control (n=7)** | **Eng-iKO$^e$ (n=6)** |
|---|---|---|
| End-systolic pressure | 27.63 ± 0.9 | **34.98 ± 2.2 (p=0.01)** |
| End-diastolic pressure | 1.42 ± 0.24 | **2.34 ± 0.3 (p=0.03)** |
| dP/dT max | 2137 ± 77 | **3354 ± 434 (p=0.017)** |

**Online Table III**

**Eng-iKO$^e$ mice have normal haematocrit and oxygen saturation one month after loss of endothelial endoglin.** Data analysed by Mann Whitney test.

| Blood parameter | Control<br>N=6 | Eng-iKOe<br>N=5 | p value |
|---|---|---|---|
| **pH** | 7.219 ± 0.01 | 7.169 ± 0.02 | 0.11 |
| **pCO2, mmHg** | 56.8 ± 2.3 | 63.5 ± 1.3 | 0.03 |
| **pO2, mmHg** | 94.75 ± 22.54 | 92.14 ± 14.89 | 0.73 |
| **Base Excess** | -4.3 ± 0.7 | -5.2 ± 1.5 | 0.83 |
| **HCO3, nmol/L** | 23.12 ± 0.7 | 23.22 ± 1.2 | 0.53 |
| **TCO2, nmol/L** | 24.83 ± 0.8 | 25 ± 1.3 | 0.57 |
| **Mean SaO2%** | 94.2 ± 2.4 | 96.4 ± 1.2 | 0.6 |
| **Na, nmol/L** | 144.2 ± 0.6 | 144.2 ± 1 | 0.92 |
| **K, nmol/L** | 4.35 ± 0.3 | 3.86 ± 0.1 | 0.14 |
| **iCa, nmol/L** | 1.31 ± 0.01 | 1.37 ± 0.01 | 0.09 |
| **Glucose, mg/dL** | 228.2 ± 7.8 | 230.2±13.1 | 1 |
| **Hct, % PCU** | 36.17 ± 0.6 | 33.6 ± 2.1 | 0.19 |
| **Hb, g/dL** | 12.3 ± 0.2 | 11.42 ± 0.7 | 0.19 |

## Online Table IV

**Incidence of Arteriovenous malformations in Control and Eng-iKO$^{e}$ mice.** Blood vessels were perfused with latex 1 week after endoglin knockdown and AVMs identified by antegrade flow of latex from arterial to venous vessels. Pelvic AVMs develop with 100% penetrance in Eng-iKO$^{e}$ mice.

| Location of AVM | Control (n=12) | Eng-iKO$^{e}$ (n=13) |
|---|---|---|
| Pubic symphysis | 0% (0/12) | 100% (13/13) |
| Gut | 0% (0/12) | 30% (4/13) |
| Thigh | 0% (0/12) | 7.6% (1/13) |
| Skin | 0% (0/12) | 0% (0/13) |

## Online table V

## Summary of Bonferroni corrected p values for two-way ANOVA analysis of aortic telemetry data in Figure 3a.

| Control vs Eng-iKOe | | | Control vs Eng-iKOe | | | Control vs Eng-iKOe | | |
|---|---|---|---|---|---|---|---|---|
| Day | Hour | p value | Day | Hour | p value | Day | Hour | p value |
| **-3** | 15-19 | 1 | **3** | 15-19 | 1 | **9** | 15-19 | 1 |
| | 19-23 | 1 | | 19-23 | 1 | | 19-23 | 1 |
| | 23-3 | 1 | | 23-3 | 1 | | 23-3 | 1 |
| | 3-7 | 1 | | 3-7 | 1 | | 3-7 | 1 |
| | 7-11 | 1 | | 7-11 | 1 | | 7-11 | 1 |
| | 11-15 | 1 | | 11-15 | 1 | | 11-15 | 1 |
| **-2** | 15-19 | 1 | **4** | 15-19 | 1 | **10** | 15-19 | 1 |
| | 19-23 | 1 | | 19-23 | 1 | | 19-23 | 0.8198 |
| | 23-3 | 1 | | 23-3 | 1 | | 23-3 | 1 |
| | 3-7 | 1 | | 3-7 | 1 | | 3-7 | 1 |
| | 7-11 | 1 | | 7-11 | 1 | | 7-11 | 1 |
| | 11-15 | 1 | | 11-15 | 1 | | 11-15 | 0.6744 |
| **-1** | 15-19 | 1 | **5** | 15-19 | 1 | **11** | 15-19 | 0.4849 |
| | 19-23 | 1 | | 19-23 | 1 | | 19-23 | 1 |
| | 23-3 | 1 | | 23-3 | 1 | | 23-3 | 1 |
| | 3-7 | 1 | | 3-7 | 1 | | 3-7 | 1 |
| | 7-11 | 1 | | 7-11 | 1 | | 7-11 | 1 |
| | 11-15 | 1 | | 11-15 | 1 | | 11-15 | 1 |
| **0** | 15-19 | 1 | **6** | 15-19 | 1 | **12** | 15-19 | 1 |
| | 19-23 | 1 | | 19-23 | 1 | | 19-23 | 1 |
| | 23-3 | 1 | | 23-3 | 1 | | 23-3 | 1 |
| | 3-7 | 1 | | 3-7 | 1 | | 3-7 | 0.0765 |
| | 7-11 | 1 | | 7-11 | 1 | | 7-11 | 0.1878 |
| | 11-15 | 1 | | 11-15 | 1 | | 11-15 | 0.4901 |
| **1** | 15-19 | 1 | **7** | 15-19 | 1 | **13** | 15-19 | 0.0754 |
| | 19-23 | 1 | | 19-23 | 1 | | 19-23 | **0.0121** |
| | 23-3 | 1 | | 23-3 | 1 | | 23-3 | **0.0045** |
| | 3-7 | 1 | | 3-7 | 1 | | 3-7 | **0.0001** |
| | 7-11 | 1 | | 7-11 | 1 | | 7-11 | 0.0894 |
| | 11-15 | 1 | | 11-15 | 1 | | 11-15 | 0.2117 |
| **2** | 15-19 | 1 | **8** | 15-19 | 1 | **14** | 15-19 | **0.0396** |
| | 19-23 | 1 | | 19-23 | 1 | | 19-23 | **0.0038** |
| | 23-3 | 1 | | 23-3 | 1 | | 23-3 | **0.0171** |
| | 3-7 | 1 | | 3-7 | 1 | | 3-7 | 0.0739 |
| | 7-11 | 1 | | 7-11 | 1 | | 7-11 | 1 |
| | 11-15 | 1 | | 11-15 | 1 | | 11-15 | 1 |
| | | | | | | **15** | 15-19 | 0.427 |

## Online table VI

## Summary of Bonferroni corrected p values for data in Figure 5

Analysis of western blot densitometry data plotted in Figure 5 C, D, F and H using two-way ANOVA to compare signalling changes with respect to baseline for each genotype, and differences between control and Eng-iKO endothelial cells at each time point. The table shows the Bonferroni corrected p values for each of these comparisons.

| | pVEGFR2/VEGFR2 | VEGFR2/tubulin | pAKT/AKT | pERK/ERK |
|---|---|---|---|---|
| **Control** | | | | |
| 0 min vs 5 min | 0.7229 | 0.582 | 1 | **0.0018** |
| 0 min vs 10 min | **0.0272** | 1 | 1 | 1 |
| 0 min vs 30 min | 0.5492 | 1 | 1 | 1 |
| **Eng-iKO$^{e}$** | | | | |
| 0 min vs 5 min | 0.5151 | 1 | 1 | 0.6972 |
| 0 min vs 10 min | 0.9369 | 1 | 1 | **7e-5** |
| 0 min vs 30 min | 0.9992 | 0.7192 | 1 | 0.1602 |
| **Control vs Eng-iKO$^{e}$** | | | | |
| 0 min | 1 | **0.0415** | 0.1144 | 1 |
| 5 min | 1 | 0.3561 | 1 | 0.5657 |
| 10 min | **0.0488** | 0.1837 | 0.0618 | **2e-5** |
| 30 min | 0.5482 | **0.0272** | 0.1327 | **0.0008** |

## References

1. Redgrave RE, Tual-Chalot S, Davison BJ, Singh E, Hall D, Amirrasouli MM, Gilchrist D, Medvinsky A, Arthur HM. Cardiosphere-derived cells require endoglin for paracrine-mediated angiogenesis. *Stem cell reports*. 2017;8:1287-1298
2. Tual-Chalot S, Mahmoud M, Allinson KR, Redgrave RE, Zhai Z, Oh SP, Fruttiger M, Arthur HM. Endothelial depletion of acvrl1 in mice leads to arteriovenous malformations associated with reduced endoglin expression. *PLoS One*. 2014;9:e98646
3. Mahmoud M, Borthwick GM, Hislop AA, Arthur HM. Endoglin and activin receptor-like-kinase 1 are co-expressed in the distal vessels of the lung: Implications for two familial vascular dysplasias, hht and pah. *Laboratory investigation; a journal of technical methods and pathology*. 2009;89:15-25
4. Mahmoud M, Allinson KR, Zhai Z, Oakenfull R, Ghandi P, Adams RH, Fruttiger M, Arthur HM. Pathogenesis of arteriovenous malformations in the absence of endoglin. *Circulation research*. 2010;106:1425-1433
5. Redgrave RE, Tual-Chalot S, Davison BJ, Greally E, Santibanez-Koref M, Schneider JE, Blamire AM, Arthur HM. Using MRI to predict future adverse cardiac remodelling in a male mouse model of myocardial infarction. *International Journal of Cardiology Heart & Vasculature*. 2016 Mar 16;11:29-34.
