# Supplementary figures and images for: Loss of Endothelial Endoglin Promotes High-Output Heart Failure Through Peripheral Arteriovenous Shunting Driven by VEGF Signaling
Source: Circ Res. 2019 Dec 6;126(2):243–57. doi: 10.1161/CIRCRESAHA.119.315974 (PMC6970547; doi:10.1161/CIRCRESAHA.119.315974)

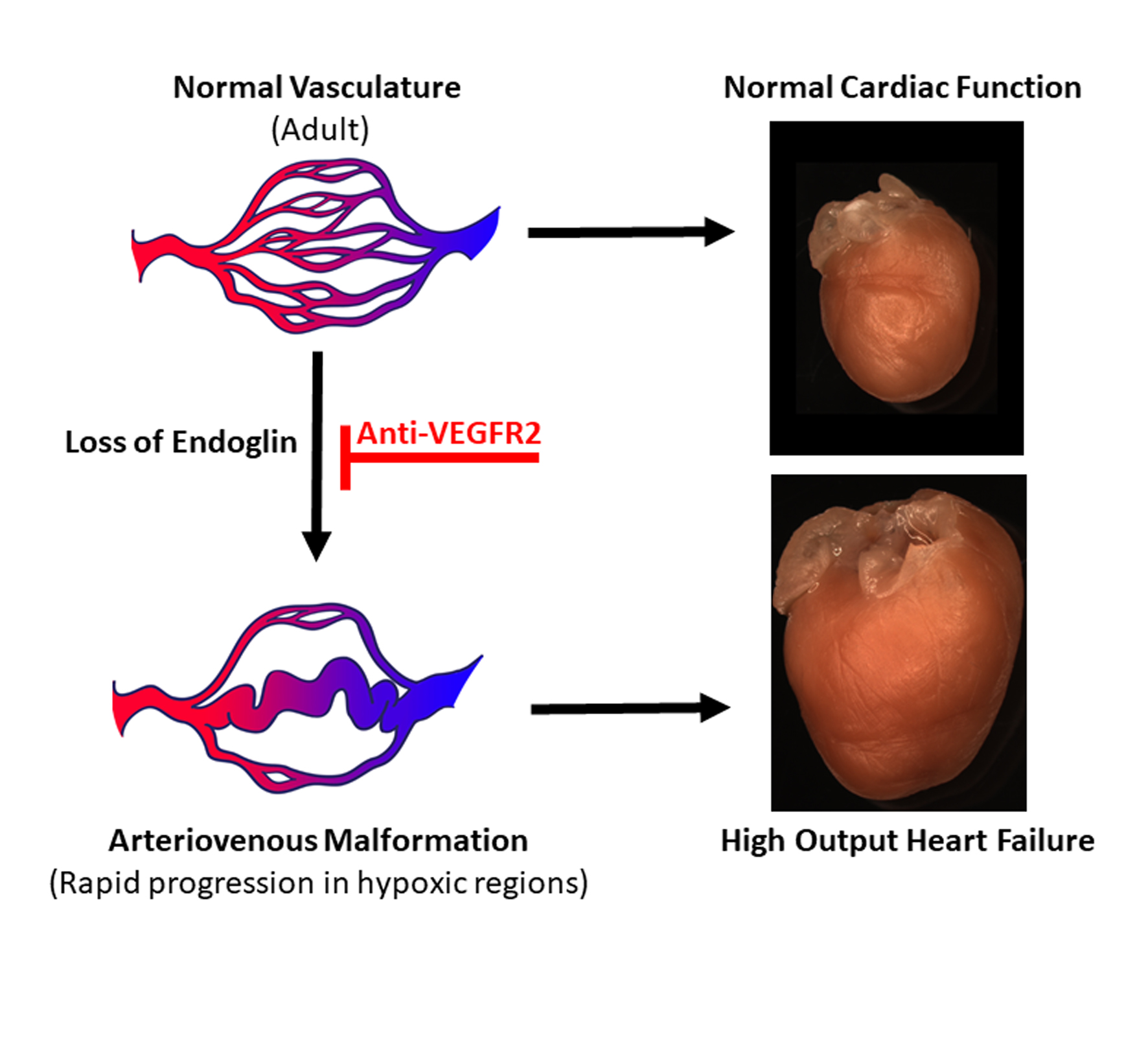

Supplement: Supplementary file 2 [file res-126-243-s002.jpg]
