# Supplementary material for: Loss of Endothelial Endoglin Promotes High-Output Heart Failure Through Peripheral Arteriovenous Shunting Driven by VEGF Signaling
Source: Circ Res. 2019 Dec 6;126(2):243–57. doi: 10.1161/CIRCRESAHA.119.315974 (PMC6970547; doi:10.1161/CIRCRESAHA.119.315974)

Original western blots.

Western Blots for Figure 2A

Full unedited gel for Figure 2A ENG

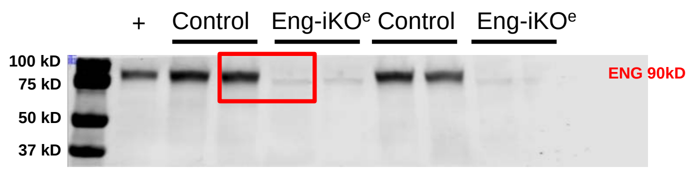

Full unedited gel for Figure 2A α-tubulin

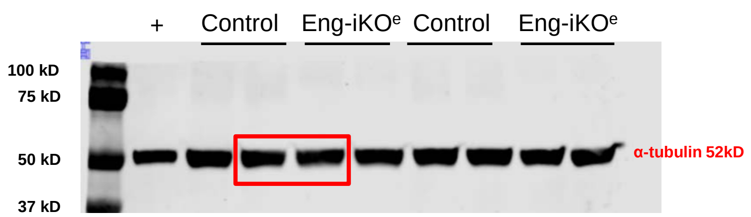

Western Blots for Figure 5:

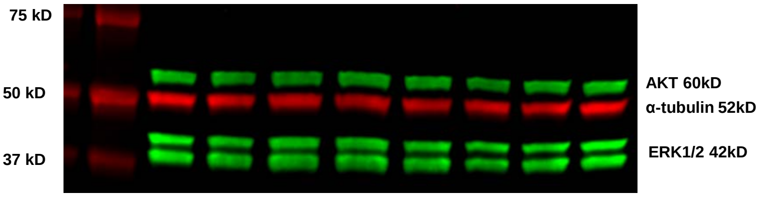

Using fluorescence filters on the LICOR system (see methods), we detected multiple proteins simultaneously with different secondary dye and altered to grayscale. Molecular weight markers are in lane 1.

Full unedited gel for Figure 5B (pVEGFR2^{tyr1173})

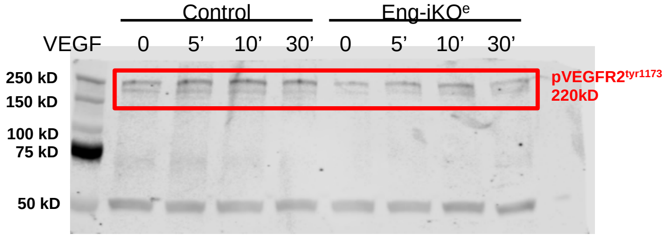

Full unedited gel for Figure 5B (VEGFR2)

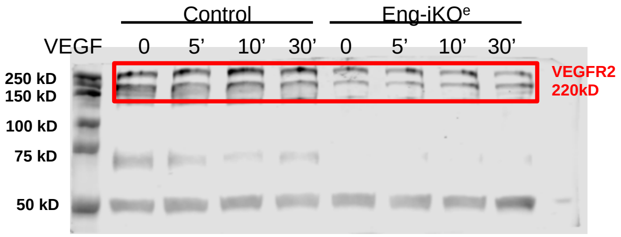

Full unedited gel for Figure 5B (α-tubulin)

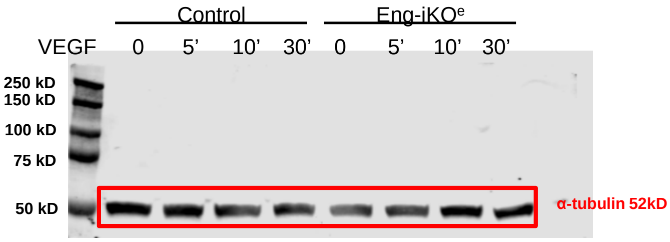

Full unedited gel for Figure 5E (pAKT^{Ser73})

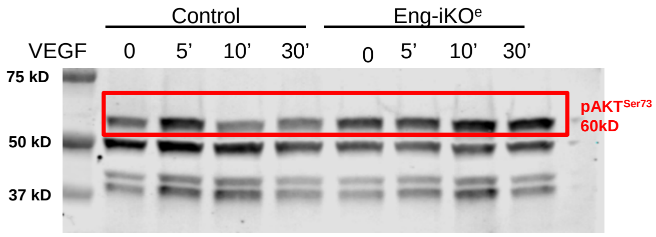

Full unedited gel for Figure 5E (AKT)

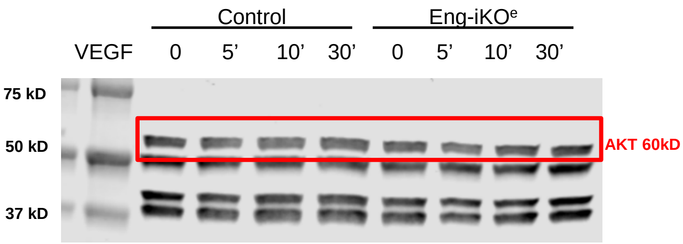

Full unedited gel for Figure 5G (pERK1/2)

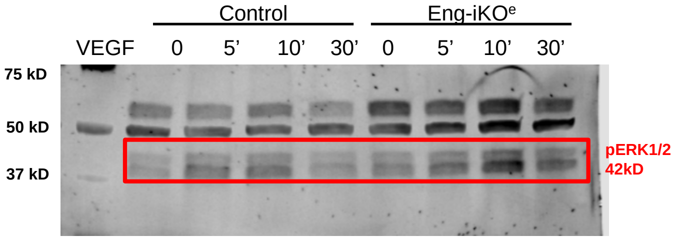

Full unedited gel for Figure 5G (ERK1/2)

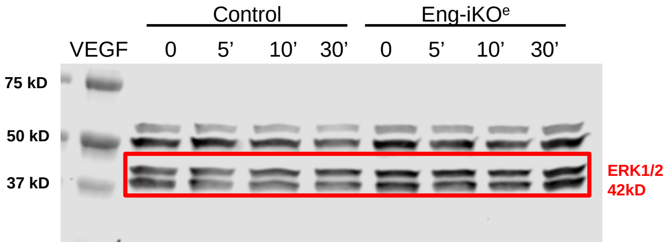

Supplement: Supplementary file 4 [file res-126-243-s004.pdf]
